# Machine learning–assisted design of a wideband Fe–SiO_2_–MXene metamaterial solar absorber for angle-insensitive thermal energy harvesting

**DOI:** 10.1038/s41598-026-57447-9

**Published:** 2026-06-10

**Authors:** Sunil Lavadiya, Vishal Sorathiya, Amar Y. Jaffar, Abdulghani Bakur Alsayegh, Khalid M. Jamil Khayyat

**Affiliations:** 1 Department of Information and Communication Technology, Marwadi University, Rajkot, Gujarat India; 2 Faculty of Engineering and Technology, Parul Institute of Engineering and Technology, Parul University, Vadodara, Gujarat India; 3 Computer and Network Engineering Department, College of Computing, Umm Al-Qura University, Makkah, 24382 Saudi Arabia

**Keywords:** Absorption, Multilayered, Metamaterial, Solar, Machine learning, Engineering, Materials science, Physics

## Abstract

The manuscript proposed an efficient broadband metamaterial-inspired multilayered solar absorber for efficient energy harvesting. Its structure is a periodic assembly of Fe, SiO₂, MXene, and Fe layers, thus facilitating the scalable fabrication. Finite element method (FEM) simulations are employed to evaluate and optimise the optical performance. The novel geometry and arrangement of the radiating elements result in peak absorptance exceeding 90% across 0.87–1.79 μm, 2.07–2.425 μm, and 3.045–3.295 μm. Parametric optimisation is conducted by varying resonator geometries (filled and solid), material combinations for resonator and ground layers (Au, Fe), and dielectric thicknesses to achieve optimal absorption characteristics. Angular stability is examined for both TE and TM polarisations over an incidence range of 0°–80°, demonstrating robust wide-angle performance. The TE and TM mode field are analysed at peak absorption wavelength. Furthermore, different machine learning models are implemented to analyse and predict absorption characteristics, validating the agreement between simulated and predicted results. The proposed design was compared and analysed with other similar works to identify performance improvements. The proposed design exhibits strong potential for broadband photothermal and solar-thermal energy harvesting applications.

## Introduction

Solar energy is among the most promising renewable energy sources due to its widespread availability, renewability, and minimal environmental impact^1^. Different ongoing solar conversion technologies, such as photovoltaic, photothermal, and hybrid systems, require high-efficiency solar absorbers. The majority of available solar absorbers have narrow spectral coverage with poor performance in the ultraviolet and near-infrared spectral regions^1,2^. Therefore, to enrich the performance of the structure achieved by the metamaterial property-based design.

These materials enable precise control over electromagnetic properties, making them highly promising for advanced applications. Their ability to manipulate electromagnetic waves empowers the creation of ultra-broadband absorbers with absorption close to unity over broad wavelength ranges. Metamaterial absorbers can be designed to achieve high absorption through optimised structural geometry and material composition, suppressing reflection and transmission, and, by doing so, delivering high absorption that is generally insensitive to polarisation variation and fluctuations in the surrounding refractive index^3,4^. Among newer materials, MXenes (two-dimensional transition-metal carbides or nitrides) are promising for plasmonic and photothermal applications. With their high electrical conductivity, intense light–matter interaction, and mechanical toughness, they are particularly interesting for visible and IR absorption. Coupled with dielectric or metallic layers, MXenes can enhance absorption, especially in the NIR region, where efficient absorption is otherwise difficult.

A tungsten–silicon dioxide ring-array metamaterial absorber: Influence of temperature on tungsten optical properties. The absorber maintained a high energy conversion efficiency, reaching 94.68% over the temperature range 473–873 K. An equivalent LC circuit model was also developed to predict magnetic polariton resonance behaviour^5^. In article^6^, a Y-shaped perfect metamaterial absorber operating in multiple bands was proposed using an FR4 substrate. The structure achieved absorption above 95% at four resonance frequencies while maintaining polarisation-independent and angularly stable performance. The absorber is suitable for EMI shielding, radar sensing, and microwave wireless communication applications. A TiN-based solar absorber with a cylindrical structure containing square holes was proposed for broadband solar-energy absorption. The absorber achieved an average absorption of 92.4% over the 300–2500 nm range and 94.8% absorption under the AM1.5 solar spectrum. Strong absorption was attributed to the combined effects of surface plasmon, guided-mode, and cavity resonances, while maintaining wide-angle and polarisation-insensitive performance^7^.

The novelty of the proposed design is as follows:

- Greater than 90% absorption achieved across visible, near-infrared, and mid-infrared wavelength regions using a Metal–SiO₂–MXene–Fe multilayer absorber. The peak absorption reaches up to 99%.
- Unequal concentric ring resonators generate multiple overlapping plasmonic resonances, resulting in wideband absorption instead of isolated narrowband peaks.
- Strong angular stability is maintained up to $\:{60}^{\circ\:}$incidence for both TE and TM modes, showing stable absorption under oblique illumination conditions.
- Combined effect of MXene-assisted plasmonic coupling and Fe back reflector produces low reflection and near-zero transmission over a broad spectral range.
- Parametric tuning of resonator dimensions and dielectric/metal layer thicknesses was carried out to obtain optimum absorption performance and resonance distribution.
- Machine-learning-based absorption prediction using AdaBoost, Gradient Boosting, KNN, Neural Network, and Random Forest models was included to compare spectral prediction capability and reduce repeated simulation effort.

The scientific contribution of the presented work introduces a broadband Metal–SiO₂–MXene–Fe absorber with concentric rings of unequal width for strong optical absorption across the visible, near-infrared, and mid-infrared regions. The combined effects of MXene-assisted plasmonic coupling, multi-resonant ring geometry, and Fe back reflection yield high absorption, low reflection, and near-zero transmission. Field analysis confirms localised electric and magnetic resonance formation at different wavelengths, while parametric studies establish the influence of resonator dimensions and layer thicknesses on spectral behaviour. The absorber also maintains stable performance up to 60° incidence under TE and TM polarisations. In addition, machine-learning-based spectral prediction was used to examine absorption behaviour using multiple regression models, supporting rapid analysis of broadband absorber performance. The article starts with the need for solar absorbers, provides a relevant literature review, and then presents the design of the proposed structure, including different optimisations and performance observations across variations. Then, using the ML model approach to compare actual and predicted values, the final performance of the proposed design is compared with that of other recent articles, with concluding remarks.

## Proposed structure design and other important considerations

A multilayer MXene-based metamaterial absorber is reported, consisting of concentric metallic ring resonators with different radii (R₁–R₄). The structure is formed as a periodic unit cell on a SiO_2_ dielectric spacer, with a metallic reflector (Fe or another suitable metal) acting as the ground plane. Rings of different sizes excite overlapping localised surface plasmon resonances (LSPRs), which leads to a wider absorption bandwidth. Periodic boundary conditions are applied to ensure scalability and uniform performance over large areas^8,9^. The absorber performance is examined for different angles of incidence and polarisation states to assess its response under practical solar and photonic conditions. Different key geometrical parameters, such as ring sizes, layer thickness, metal type, and the position of the MXene layer, are modified to improve electromagnetic-field confinement and broaden the absorption range. This study supports existing design approaches for MXene-based metamaterial absorbers and indicates their suitability for broadband energy harvesting and photonic sensing applications.

The normalised impedance $\:{Z}_{eff}$ is obtained by Eq. (1)^10^.

1 $$\:{Z}_{eff}=\sqrt{\frac{{\left(1+{S}_{11}\right)}^{2}-{S}_{21}^{2}}{{\left(1-{S}_{11}\right)}^{2}-{S}_{21}^{2}}}$$

The complex refractive index * n* is obtained from the measured phase of the transmission through a thickness * d,* calculated using Eq. (2).

2 $$\:n=\frac{-\left({e}^{in{k}_{0}d}\right)\:}{{k}_{o}d}$$

3 $$\:{e}^{in{k}_{0}d}=X\pm\:i\sqrt{1-{X}^{2}}$$

4 $$\:X=\frac{1\:}{2{S}_{21}\left(1-{S}_{11}^{2}+{S}_{21}^{2}\right).}$$

5 $$\:{\mu\:}_{r}=n{Z}_{eff}\:,\:{\epsilon\:}_{r}=\frac{n}{{Z}_{eff}}$$

where $\:{k}_{0}$ is the free-space wavenumber. To evaluate the term $\:{e}^{in{k}_{0}d}$ from the S-parameters. The relative permeability ($\:{\mu\:}_{r}$) and permittivity ($\:{\epsilon\:}_{r}$) of the absorber are derived from the effective refractive index (* n*) as per Eq. (3) and impedance ($\:{Z}_{eff}$) using the standard Nicolson–Ross–Weir relations^11,12^. Here, $\:{S}_{11}$and $\:{S}_{21}$are reflection and transmittance coefficients.

The proposed absorber was analysed using the frequency-domain solver available in COMSOL Multiphysics over the wavelength range of 0.3–3.3 μm. To accurately capture the narrow plasmonic resonances generated by the concentric metallic rings and by MXene-assisted coupling, dense spectral sampling was used throughout the simulation domain. A total of 1500 spectral sampling points were considered across the investigated wavelength range. The selected sampling density provided sufficient resolution of the resonance dips, absorption peaks, and oscillatory behaviour observed in the near-infrared and mid-infrared regions. For electromagnetic discretisation, COMSOL’s physics-controlled tetrahedral meshing scheme was used, with additional local mesh refinement near the metallic ring resonators, the MXene interface, and the dielectric boundaries. These regions contain strong field localisation and rapid phase variation; therefore, finer elements were necessary to accurately resolve the plasmonic response.

The maximum mesh element size was selected to remain substantially smaller than the effective wavelength inside the dielectric medium. Extremely fine meshing was applied around the ring edges and narrow curved sections where strong electric-field concentration was observed. The final mesh contained approximately $\:1.2\times\:{10}^{6}$ domain elements after adaptive refinement. Mesh convergence analysis was carried out systematically by gradually increasing the mesh density and comparing the resulting absorption spectra. Periodic boundary conditions were assigned along the lateral x- and y-directions to model the infinite periodic array of the unit cell. Perfectly matched layers (PMLs) were applied along the propagation direction to suppress artificial reflections from the simulation boundaries. The incident electromagnetic wave propagated normally along the z-axis under both TE and TM polarisations.

**Fig. 1:**
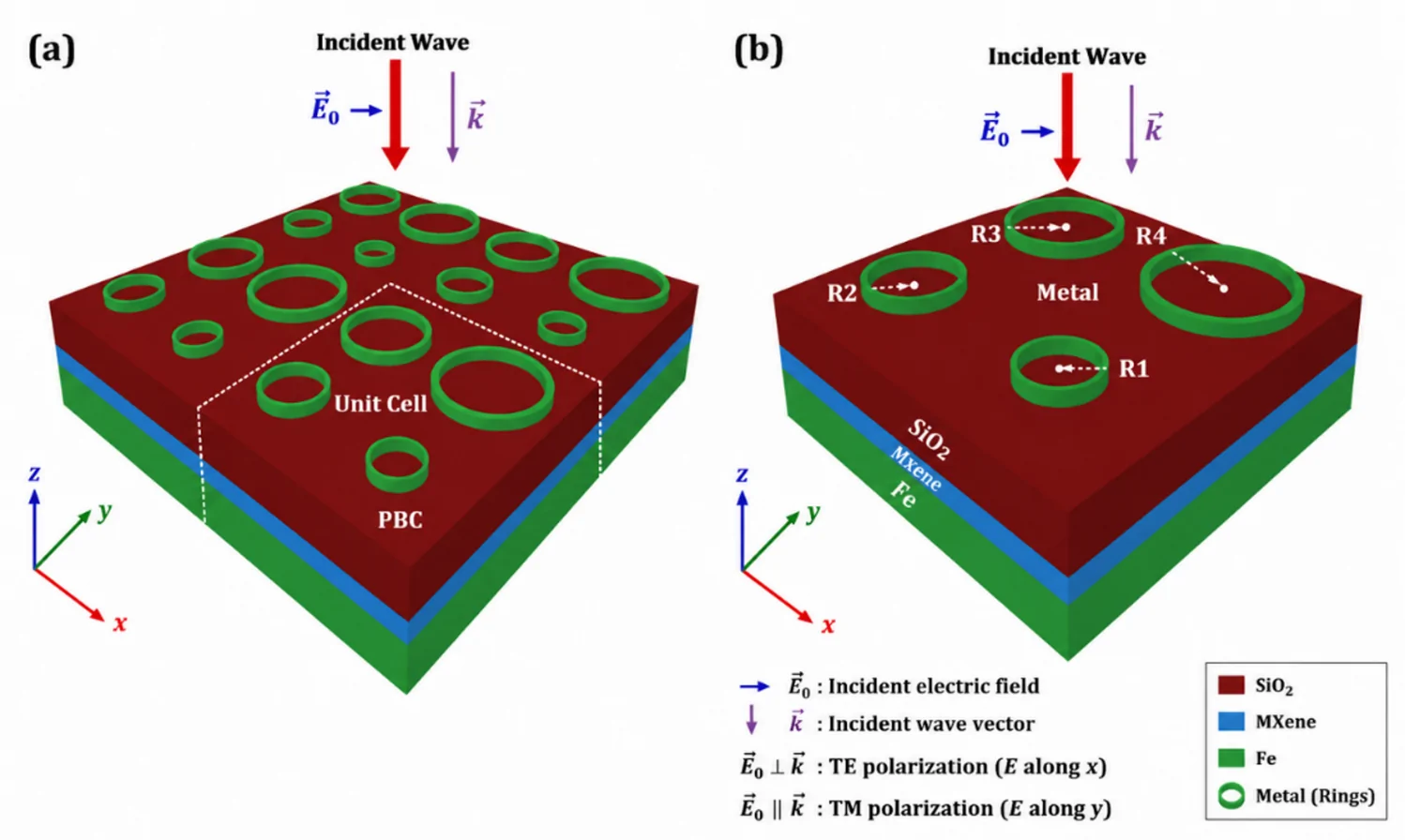
(a) Unit cell of the periodic metamaterial with concentric circular resonators with PBC. (b) Enlarged view showing four resonator radii (R_1_–R_4_) on the metal layer over SiO₂/MXene/Fe stack under the incident wave.

In Fig. 1(a), a 3D view of the periodic unit cell of the solar absorber is shown, where PBC (Periodic Boundary Condition) denotes the periodicity in the x–y plane, enabling uniform, continuous absorption over a large surface area. The metallic circular ring resonators, having different inner and outer radii, are spread across the surface to absorb a broad spectral range of incident light. The sectional view of the absorber, along with the multilayer configuration and its interaction with incident electromagnetic waves, is shown in Fig. 1(b). The varying sizes of the circular resonators (R_1_–R_4_) enable multi-resonant absorption over a broad wavelength range (UV–NIR). The topmost metal ring layer is essential for generating localised surface plasmon resonances (LSPRs)^13^. A change in ring size creates several resonance peaks, thereby maximising absorption and extending it into the ultrawideband regime. The layer primarily serves to effectively trap incident light and convert it into localised electromagnetic energy.

The SiO₂ dielectric spacer layer provides impedance matching between the metal resonators and the substrate, reducing reflection loss and enhancing light–matter interaction^14–16^. The SiO₂ layer also acts as an optical cavity, creating constructive interference to further enhance absorption. Underneath the SiO₂ layer, the MXene interlayer is an electrically conductive and plasmonic platform. MXenes, with their electrical conductivity and surface tunability, enhance hole-electron generation and thermal dissipation, thereby improving device stability and absorption efficiency^17–19^. MXenes support the excitation of surface plasmon polaritons (SPPs), which interact with the localised surface plasmon resonances (LSPRs) in the top ring structures, resulting in much stronger electromagnetic field confinement^20,21^. The Fe back reflector is found to play an important role, as the unabsorbed photons are reflected back into the structure, thereby improving light trapping and reducing transmission losses, and, in this way, near-unity absorption is achieved.

The resonator layer is a vital part in the design as it produces LSPRs that are mainly responsible for the broadband absorption characteristics. As depicted in Fig. 1(b), it consists of four ring resonators with different radii (R1–R4) in a single periodic unit cell. The differences in the sizes of the rings create multiple resonance modes, allowing light of different wavelengths to be absorbed by each resonator. This multi-resonant coupling leads to enhanced absorption over a broad spectral range. The hollow-ring geometry supports both inner- and outer-surface plasmon modes, resulting in stronger electromagnetic-field confinement compared to solid circles. The periodic arrangement and PBCs ensure that uniform absorption is preserved even over large areas, without significantly losing its efficiency. By optimising the resonator dimensions, spacings, and suitable materials, effective impedance matching with free space can be realised, thereby enhancing light-matter interaction. The structure lends itself to use as a solar energy harvester, and in photodetectors and optical sensors. Thus, the Metal–SiO2–MXene–Fe multi-layer stack provides an ultra-efficient absorber, enabling near-perfect absorption across the broadband spectral range of 0.3–3.3 μm. The multi-resonator-based absorber offers broadband, polarisation-independent, and angle-insensitive absorption. As such, it is suitable for solar energy conversion, photothermal and infrared detection.

Experimental validation of the proposed Metal–$\:{\mathrm{S}\mathrm{i}\mathrm{O}}_{2}$–MXene–Fe broadband absorber can be performed through a sequence of nanoscale fabrication, structural characterisation, and optical measurement procedures. Since the designed structure consists of multilayer thin films and concentric metallic resonators operating in the visible and infrared region, precise control over thickness, geometry, and material uniformity is necessary to reproduce the simulated absorption behaviour experimentally.

The fabrication process may begin with the preparation of a clean substrate, such as silicon, quartz, or sapphire, because these substrates provide good thermal stability and smooth surfaces for thin-film growth. Initially, the Fe back reflector layer can be deposited using magnetron sputtering or electron-beam evaporation. The thickness of this layer should remain sufficiently larger than the optical skin depth so that electromagnetic transmission becomes nearly zero throughout the operating wavelength region. During deposition, parameters such as chamber pressure, deposition rate, and substrate temperature should be carefully controlled because the optical loss and surface roughness of the Fe layer directly affect the absorption response.

After deposition of the Fe reflector, the $\:{\mathrm{S}\mathrm{i}\mathrm{O}}_{2}$ A dielectric spacer layer can be formed using plasma-enhanced chemical vapour deposition (PECVD), radio-frequency sputtering, or atomic layer deposition (ALD). The dielectric thickness must closely match the optimised simulated value, as the spacer controls the cavity resonance conditions and electromagnetic confinement between the resonator layer and the metallic reflector. Even small deviations in dielectric thickness can shift the resonant wavelengths and alter the impedance-matching condition.

The MXene layer can then be integrated above the dielectric spacer. The MXene nanosheets may be synthesised through selective that followed by exfoliation and delamination. The prepared MXene solution can be deposited using spin coating, spray coating, or vacuum-assisted filtration. Uniformity of the MXene layer is particularly important because its conductivity and surface-plasmon behaviour strongly contribute to optical absorption and resonance damping. Post-deposition annealing under controlled temperature conditions may also be required to improve film adhesion and electrical conductivity. Following the multilayer preparation, the top metallic concentric ring resonators can be fabricated using electron-beam lithography (EBL), nanoimprint lithography, or focused ion beam (FIB) patterning. Electron-beam lithography is preferred because the resonator dimensions are in the nanometer scale and require high-resolution pattern definition. In this process, a resist layer is first spin-coated onto the MXene surface, followed by electron-beam exposure according to the designed concentric ring geometry. After the resist pattern is developed, the Au resonator layer can be deposited by thermal evaporation or sputtering, and the unwanted metal regions can then be removed by lift-off, leaving the patterned concentric rings on the surface.

Then the dimensional accuracy and material quality of the structure should be examined carefully. Scanning electron microscopy (SEM) can be used to verify the ring dimensions, periodicity, and fabrication accuracy of the unit-cell geometry. Atomic force microscopy (AFM) may be used to measure surface roughness and uniformity of layer thickness. Elemental analysis through energy-dispersive X-ray spectroscopy (EDS) can confirm the presence of Fe, Au, $\:{\mathrm{S}\mathrm{i}\mathrm{O}}_{2}$, and MXene-related elements, while X-ray diffraction (XRD) analysis may be used to examine crystallinity and structural stability of the deposited films. The optical characterisation of the fabricated absorber can be carried out using UV–Visible–Near-Infrared (UV–Vis–NIR) spectrophotometry and Fourier-transform infrared spectroscopy (FTIR). Reflectance measurements should be performed over the wavelength range of 0.3–3.3 μm under normal and oblique incidence conditions. Since the Fe back reflector suppresses transmission almost completely, the absorptance can be determined directly from the reflection spectrum.

To further examine the physical origin of the resonances, near-field optical characterisation methods such as near-field scanning optical microscopy (NSOM) or infrared nano-imaging may be employed. These techniques enable visualisation of localised electromagnetic-field confinement around the edges of the metallic ring and at dielectric interfaces. Such measurements would provide direct confirmation of the LSPR and magnetic resonance effects predicted in the simulation study. Thermal stability analysis can also be carried out by measuring the absorption response at elevated temperatures, as MXene and Fe layers may exhibit variations in conductivity with temperature. Repeated optical measurements before and after thermal cycling would help evaluate structural reliability and long-term operational stability.

### Performance analysis and observations

The proposed absorber operates over a broad optical region extending from 0.3 to 3.3 μm, covering the visible, near-infrared, and part of the mid-infrared spectrum. Within this overall region, the absorption response contains several continuous high-absorption bands rather than a single uninterrupted flat band. From the obtained results, absorptance remains above 90% over the wavelength intervals 0.87–1.79 μm, 2.07–2.425 μm, and 3.045–3.295 μm. The corresponding average absorptance values over these regions are approximately 95.8%, 93.4%, and 94.1%, respectively. When the complete 0.3–3.3 μm spectrum is considered, the average absorptance remains around 89%, indicating strong broadband optical absorption over a wide spectral interval.

**Fig. 2:**
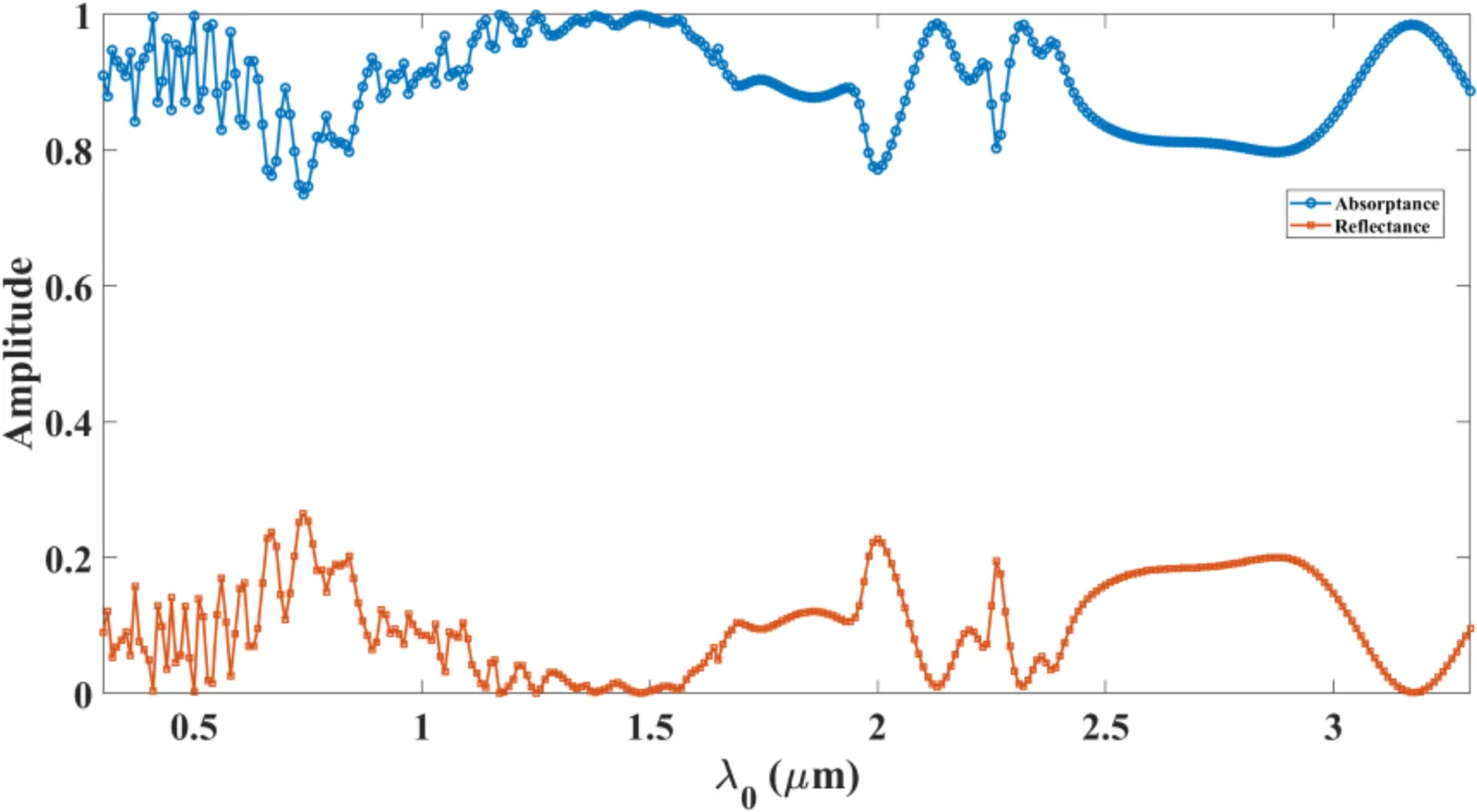
Absorption and reflection spectra of the proposed structure over the 0.3–3.3 μm wavelength range, showing high absorptance and low reflectance across a broad spectral region.

The combined effect of multiple resonance modes excited by the different-sized circular resonators is responsible for the broadband behaviour. Multiple overlapping high-absorption regions occur throughout the spectrum due to resonant light absorption. For the oscillations and local absorption dips observed in Fig. 2, resonant interactions among the metallic rings, the dielectric spacer, the MXene layer, and the Fe reflector were found. Due to the presence of resonators of different dimensions in the structure, the localised surface plasmon modes are not confined to a single wavelength. When they interact, they exhibit constructive and destructive interference, causing small fluctuations in the absorption curve. Single-resonant metasurface absorbers have been developed to cover a broad spectrum of optical frequencies and to exhibit the characteristics of thin, high-contrast, and broadband absorbers. For multi-resonant metasurface absorbers operating over a wide optical window, such variations are expected.

The low reflection response observed across most of the wavelength range confirms that the structure maintains good impedance matching with free space over several spectral regions. The $\:{\mathrm{S}\mathrm{i}\mathrm{O}}_{2}$ spacer and MXene layer contribute to field confinement and optical loss, while the Fe back reflector suppresses transmission and increases photon recirculation inside the multilayer stack. As a result, strong absorption is maintained even though the spectral response contains multiple resonance features instead of a single smooth table^22,23^. Although the absorption spectrum contains several oscillatory features, the structure maintains high average absorptance over broad visible and infrared wavelength intervals due to the superposition of multiple resonance modes generated by the differently sized ring resonators. The broadband response, therefore, originates from combined multi-resonance absorption rather than a single continuous resonance band.

**Fig. 3:**
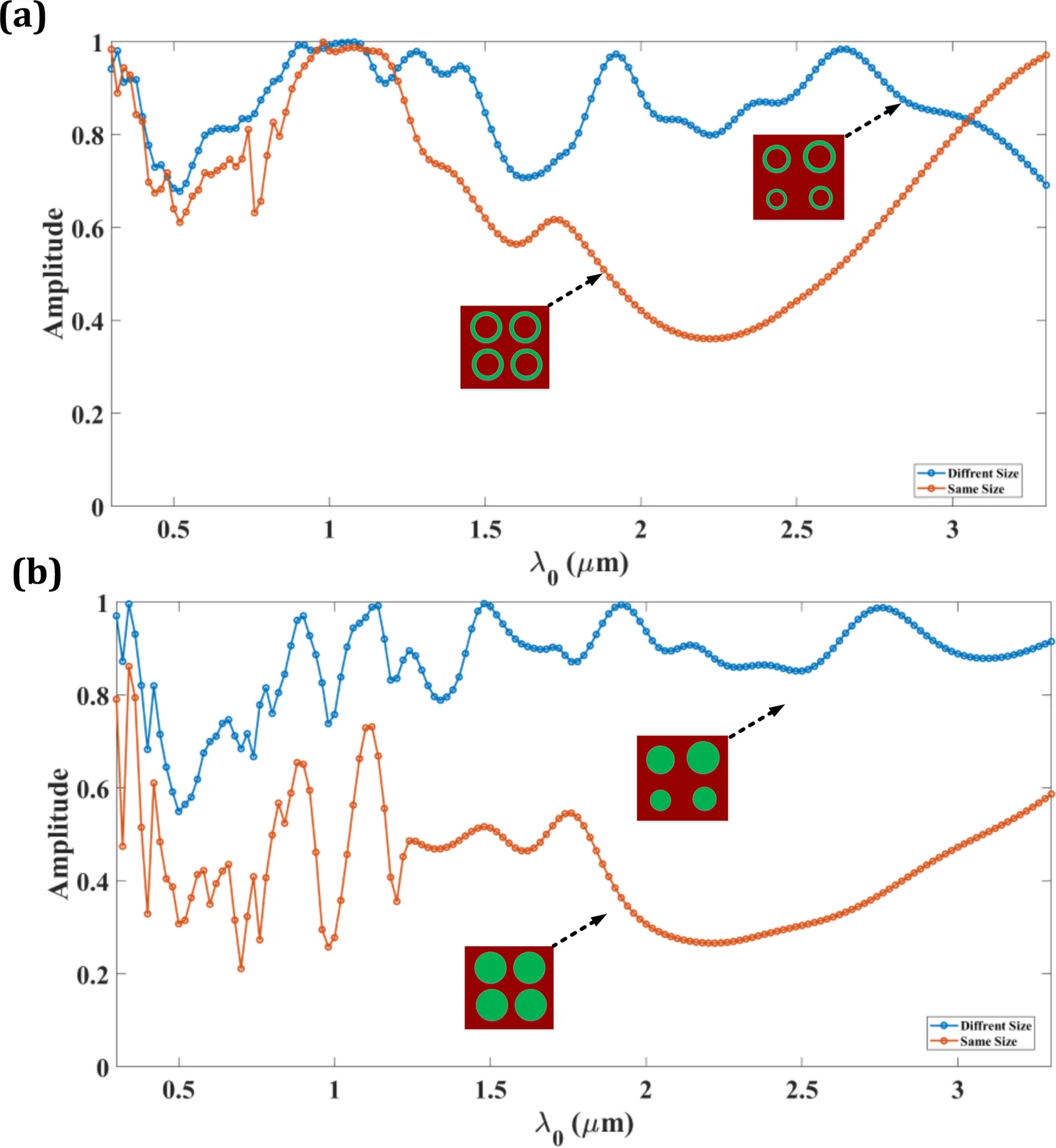
Comparison of absorption responses for (a) ring-shaped (hollow) and (b) solid circular resonator arrays, using identical and varied resonator sizes, over the wavelength range of 0.3–3.3 μm.

Figure 3 shows the absorption response of two plasmonic absorber configurations. The ring-shaped (hollow) and solid circular resonators are shown in Fig. 3(a) and (b), respectively. In both configurations, resonator designs of the same size and different sizes are considered. The blue curve is a configuration of different-sized elements, and the orange curve is a design of a number of the same-sized elements. In the visible range to near-infrared, as observed in Fig. 3 (a), the stronger and wider absorption can be obtained through the ring-type resonators. The simultaneous confinement of plasmonic modes at the inner and outer edges of the rings is the principal cause for this behaviour. Multiple localised surface plasmon resonances (LSPR) are excited, promoting broadband absorption across a range of ring dimensions. This single-size ring configuration results in low-resonance amplitude modes, leading to a narrow absorption bandwidth. As shown in Fig. 3(b), the solid circular resonators exhibit weak absorption due to the absence of an inner cavity, which limits the potential field confinement. With different element sizes, absorption improves due to overlapping resonances, which broaden and stabilise the absorption behaviour. The inserted figures show the corresponding geometrical configurations and corroborate the role of resonator geometry and size in light–matter interactions. The different dimensioned hollow resonators exhibit superior broadband absorption and plasmonic coupling compared to the solid circular configuration with uniform dimension.

**Fig. 4:**
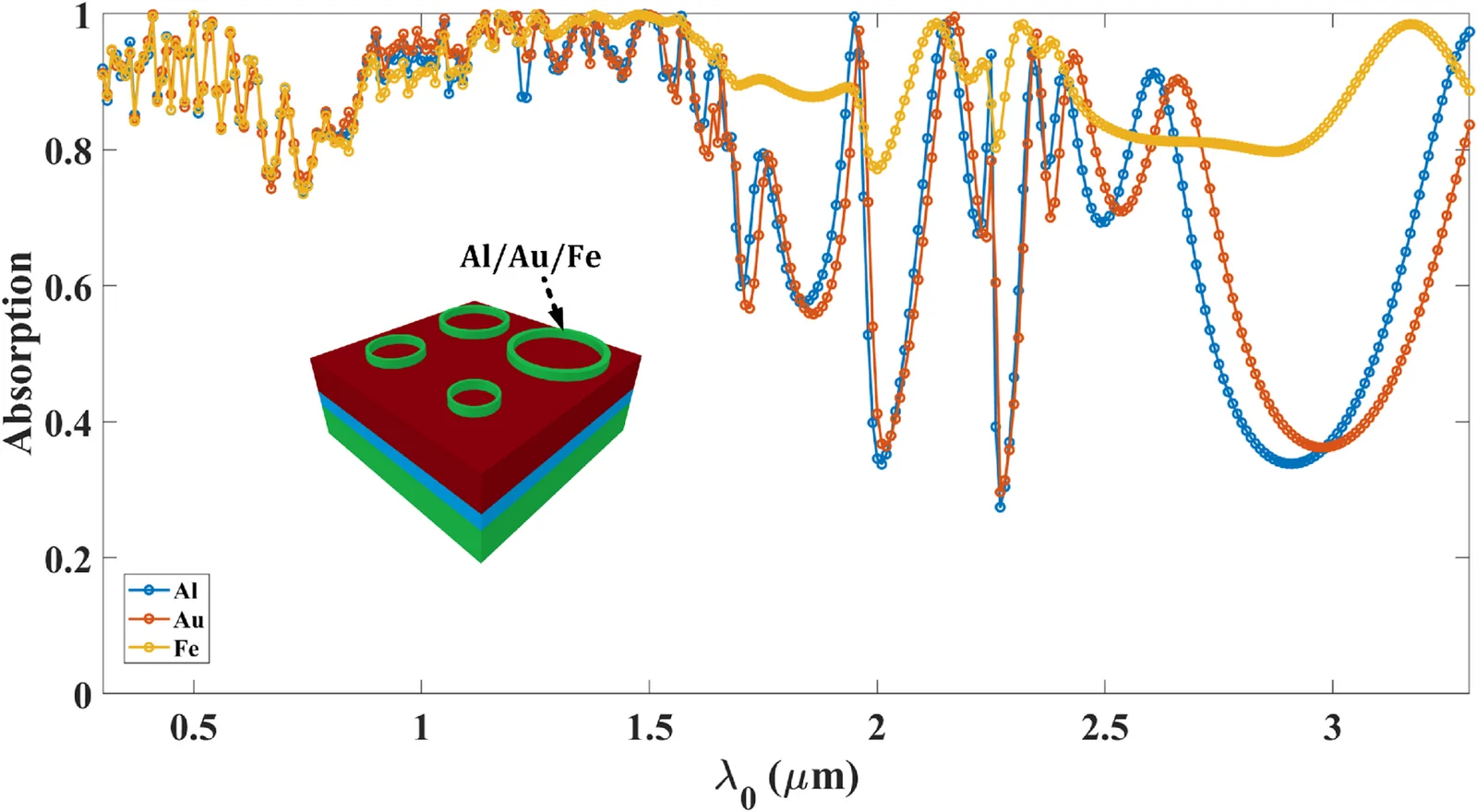
The absorption spectra for structures with various metallic layers (Al, Au, and Fe), emphasising how the metal choice influences resonance behaviour, with Fe exhibiting relatively higher broadband absorption.

Figure 4 presents the absorption spectra of the absorber with different reflector metals; i.e. three absorber structures on the top of which Al, Au and Fe are added as back reflector. The structure with circular resonators and a metallic reflector layer was simulated. All three magnets exhibit high absorptivity in the visible range (0.4–0.7 μm), with values exceeding 90%. Thus, the light field is largely confined, resulting in low reflection loss. In the NIR-position (0.7–1.5 μm), the Fe-based structure shows an almost stable high absorption response over the full range. In comparison, the Al- and Au-based absorbers exhibit a more oscillatory absorption spectrum with several dips due to resonant effects. Thus, Al and Au offer less uniformly favourable absorptivity in the NIR-position compared to Fe.

The proposed structure shows more than 90% absorption over 0.87–1.79 μm, 2.07–2.425 μm, and 3.045–3.295 μm. This improved behaviour is related to a relatively large refractive index of Fe, its own optical losses, and the strong confinement of incident light within the multilayer structure, which together lower transmission and enhance light trapping. By comparison, Al- and Au-based structures exhibit sharper resonance dips and an observable reduction in absorption beyond 2.8 μm, and thus are not useful in the mid-infrared spectrum. However, the Fe-based design maintains the high absorption to about 3 μm, which implies that it can preserve the near-complete absorption in a broad wavelength range. These findings show that Fe is a better resonator material than Al and Au and can hence be used in ultrawideband solar absorbers and photothermal energy conversion use.

**Fig. 5:**
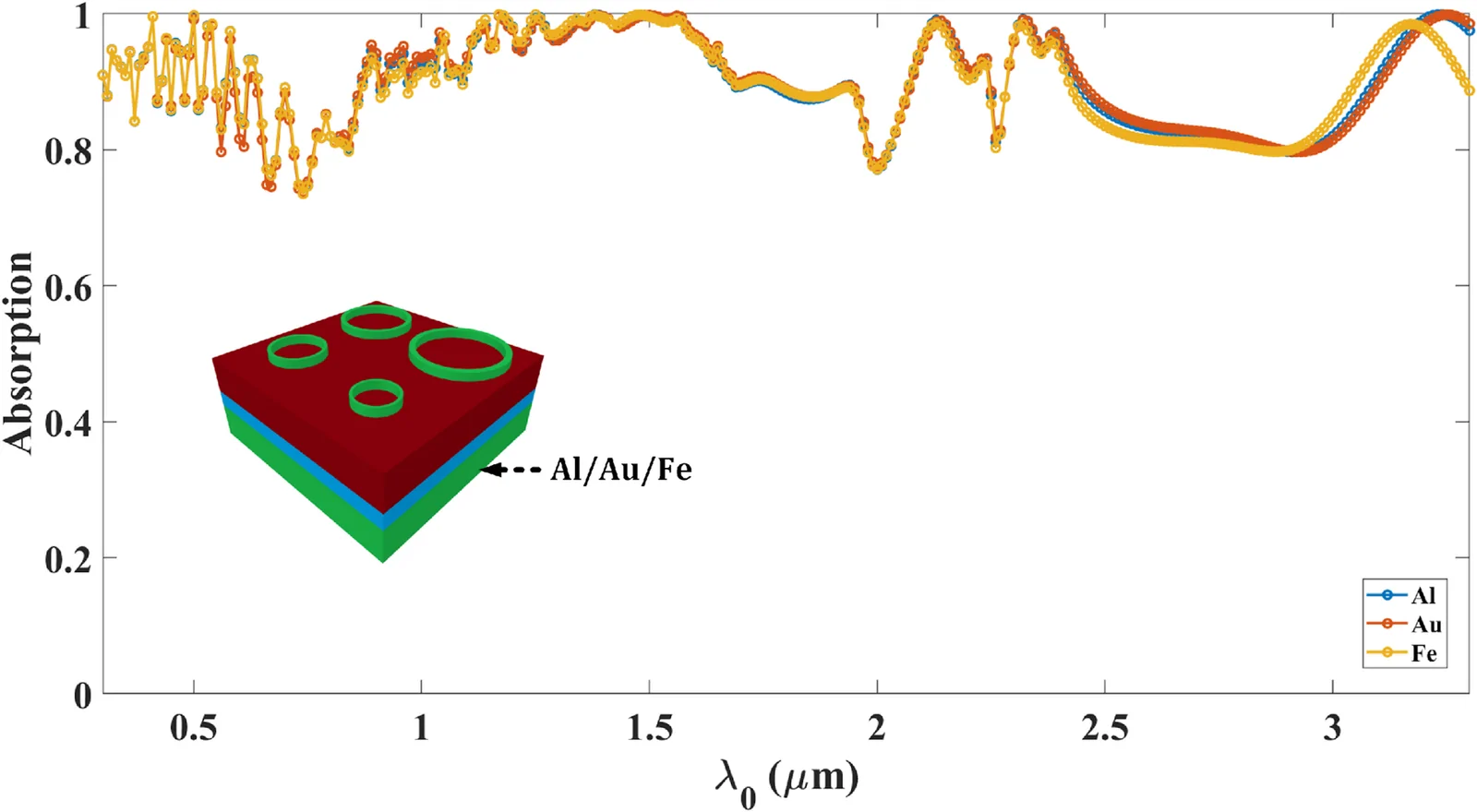
Absorption response for different metals (Al, Au, Fe).

Figure 5 shows the simulated absorption spectra of multilayer absorber structures in which Al, Au, and Fe are used as back-reflector metals, as shown in the inset. In the visible wavelength region (0.4–0.7 μm), almost complete absorption is observed for all three structures, indicating strong electromagnetic confinement and very low reflection. When the wavelength is increased into the near-infrared region (0.7–1.5 μm), the absorption remains above 85% in each case, suggesting that resonant interactions within the multilayer stack are maintained. For wavelengths longer than 1.5 μm, periodic variations begin to appear in the absorption spectra. These variations are basically attributed to coupled plasmonic modes in tandem with Fabry -Péret type cavity resonances formed between the various interfaces of the multilayer structure.

The spectral behaviour of all three metals is nearly similar, indicating that the structural design is the dominant contributor to broadband absorption, rather than the metal of the reflector. Nevertheless, the design containing Fe at the back exhibits slightly more coherent absorption across the entire wavelength spectrum. It is possible to attribute this behaviour to the natural material losses of Fe and to its capacity to trap and dissipate the incoming energy in the cavity, thus reducing the transmission. Minor disparities are observed in the mid-infrared, where Al and Au exhibit slightly lower absorption than Fe. The nearly flat absorption plateau from 0.5 μm to 1.5 μm also indicates that the optical response is constant over a broad spectral range.

**Fig. 6:**
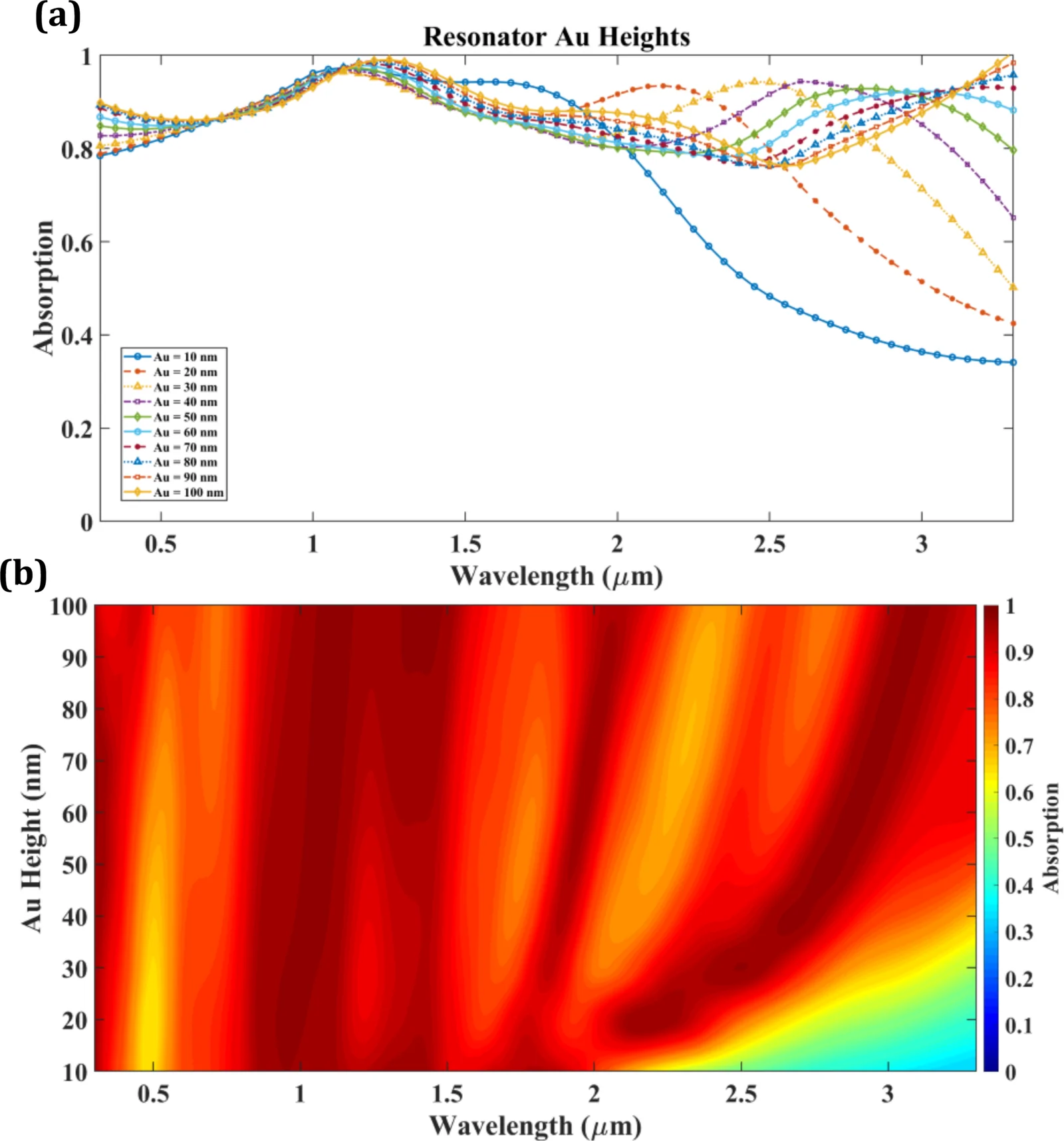
(a) The absorption spectrum of gold resonators of various heights (10 to 100 nm). The resonance behaviour is height-dependent, with an absorption constant throughout the spectrum. (b) A contour map of levels of absorption conditional on gold height and wavelength, indicating areas of maximum absorption and broadband response.

The variation in the height of the gold (Au) resonator influences the absorption properties of the multilayer absorber, as illustrated in Fig. 6. Figure 6(a) is the simulation of the absorption spectrum of the Au resonator heights of 10 nm to 100 nm. The colour map is shown in Fig. 6(b) and shows the absorption intensity versus wavelength and height of Au.

**Fig. 7:**
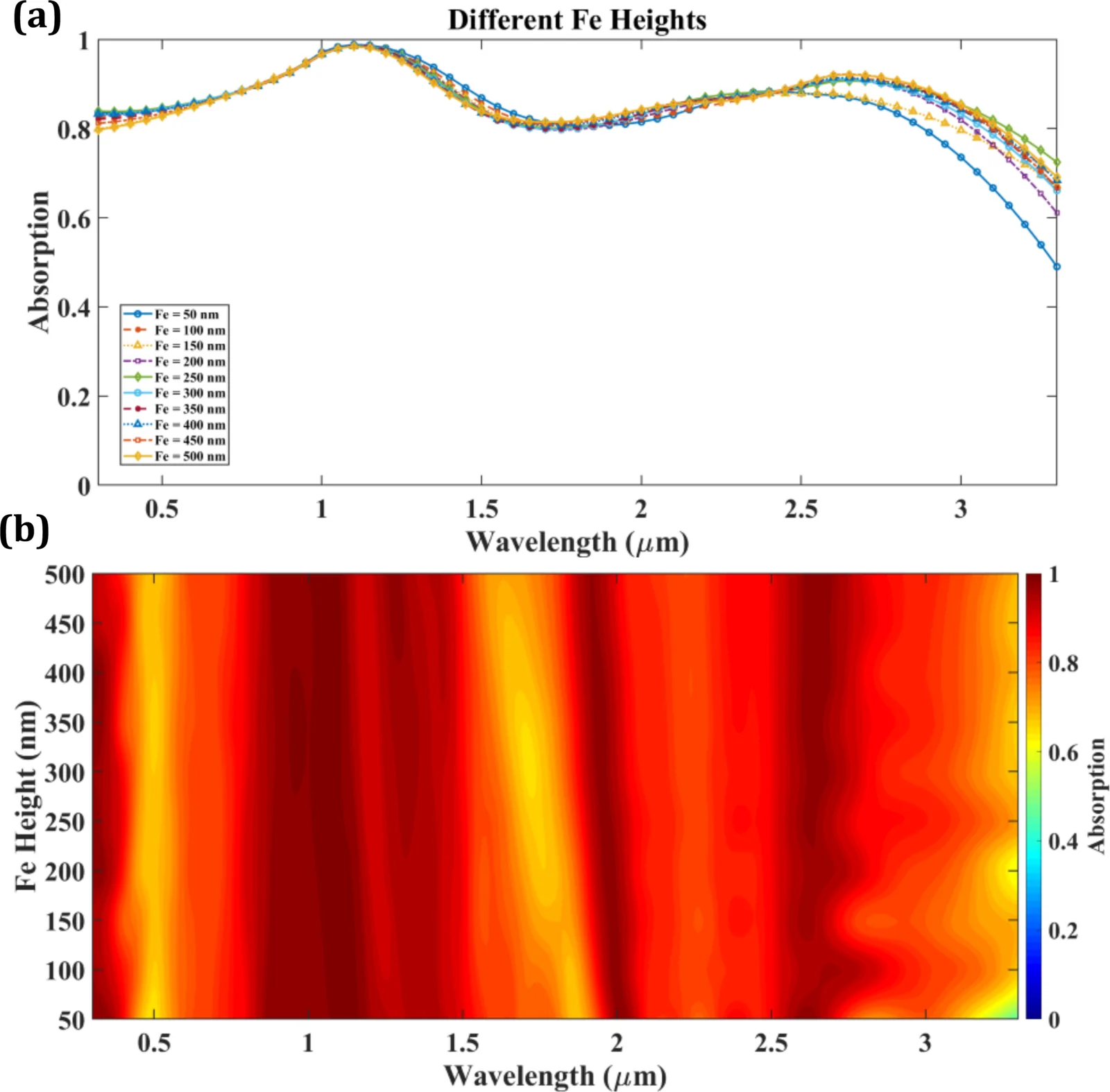
Absorption spectra with different layer heights of Fe (50 to 500 nm). (a) Line plot (b) Contour plot.

The resonator heights are maintained at 60–90 nm, keeping absorption above 85 per cent across a wide range of wavelengths, indicating good coupling between the cavity modes and the localised surface plasmons. The absorption behaviour is most stable at Au heights of about 70–80 nm, with an absorption rate greater than 80% up to an almost 3 μm. This tendency is evident in Fig. 6(b) as broad red areas across the visible and near-infrared regions. At a lower Au layer thickness (10 nm), the absorption region becomes smaller and less effective. Conversely, the homogeneous broadband response is a little disturbed by the thicker layers on top of 90 nm. Such findings imply that the height of the Au resonator must be controlled precisely to achieve high, wide-band plasmonic absorption.

Figure 7 indicates the variation in the absorption behaviour with the change in the thickness of the Fe (iron) layer in a wide wavelength range. Figure 7(a) shows absorption spectra of Fe heights at a step size of 50 nm until the 500 nm mark. All curves are associated with a dissimilar Fe thickness. Absorption is high at lower wavelengths, principally below 1 μm, and there is a peak near 1.3 μm. The absorption then decreases slowly, starting at 3.2 μm, with some slight variations in the curves. Absorption remains relatively high across most of the spectrum; even slight variations can be observed as the Fe height varies. Layers of Fe that are 50 –150 nm thick exhibit a marginally lesser absorption at approximately 2.5 μm relative to those of greater thickness, like 400 nm and 500 nm. This behaviour will be associated with thickness-based interference within the structure. Figure 7(b) 2D colour map can be used to visualise this effect with red and yellow areas representing high absorption, and blue areas a weaker response. Repeating patterns of the bands can be observed between 1.2 μm and 2.8 μm, indicating that the resonance changes as the Fe layer thickness increases. Such behaviour resembles Fabry -Pérot cavity effects. Absorption is high below 1 μm in all values of Fe thickness. The reduction in depth was observed across the thickness range of 200 nm to 350 nm.

**Fig. 8:**
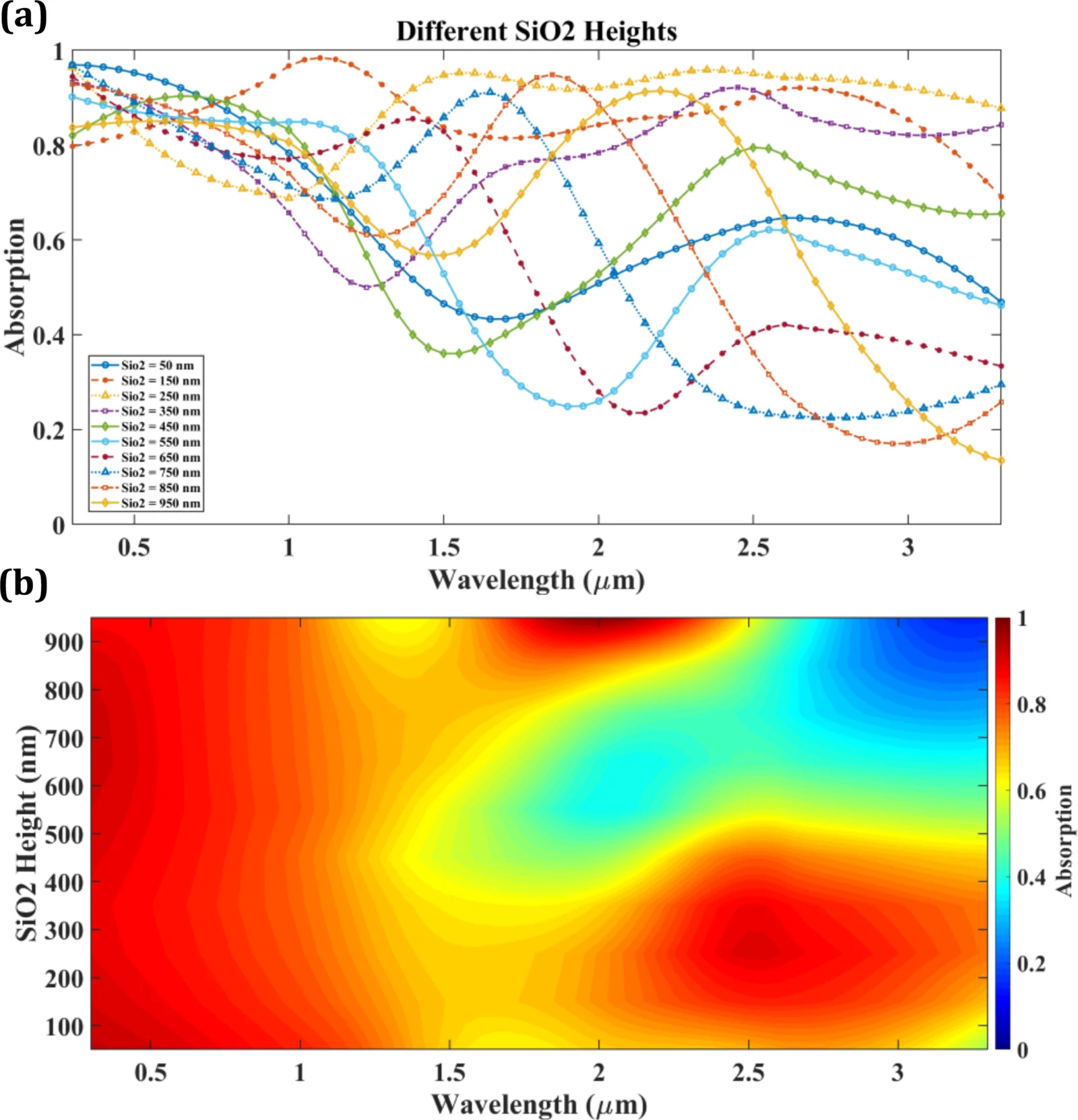
Absorption spectra of different heights (50 to 950 nm) of the spacer SiO_2_.

Figure 8 checks the size of the SiO 2 spacer thickness on the absorption. Figure 8(a) shows the curves of absorption at spacer thickness between 50 nm and 950 nm. It is observed that the curves begin to exhibit wave-like variation, and this variation is more pronounced as the spacer layer thickness increases. This is because the linen is much more extended within the structure, and the interference is more within the cavity. When the thickness is varied, the peaks and dips shift, indicating that the resonance is size dependent. This effect is also illustrated in the contour plot (Fig. 8(b)), where bands of high and low absorption are evident, with the predominant region occurring between 1 and 3 μm. These bands are connected with interference within the structure. The absorption remains high for most spacer thicknesses below 1 μm. As the wavelength increases, absorption varies less uniformly, indicating that thickness affects a person more in this region.

The optical response is dominated by behaviour similar in the two plots, suggesting that it is governed by the thickness of the dielectric layer. A change in the SiO₂ thickness is sufficient to adjust the absorption level. Switchable devices such as absorbers, sensors, and filters in the near- and mid-infrared. With proper choice of dielectric thickness, absorption can be increased or reduced at certain wavelengths due to cavity resonance effects. Because of this, the structure can be used when employing wavelength-selective applications. The novelty of structure is not only in terms of a single parameter, but also in terms of the implementation of MXene materials itself. The contribution is the combined action of the multilayer structure, resonator structure, broadband optical response, polarisation properties, and the optimisation method applied in the design.

The reported absorber structures used in the previous studies rely largely on discrete patch structures, periodic square resonators or single-sized metal components. In many of these, the absorption band is very narrow, covers only a small range in the infrared spectrum, or varies strongly with polarisation and incidence angle. High absorption is also achieved in some reported structures, which must be constructed with complex multi-layer designs or very tight fabrication tolerances. The present design, however, comprises multiple concentric circular resonators of different sizes and a Metal-SiO2-MXene-Fe arrangement, which enables multiple resonance modes to coexist within a single unit cell.

The functions of each of the parts proposed in the structure are diverse and complementary:

- Multiple localised resonance modes over multiple wavelength regions are created by the concentric ring resonators of different radius. They have a spectral overlap to give the broadband response.
- Infrared plasmon-assisted absorption and additional optical loss are introduced with the MXene layer.
- SiO2 spacer is used for field confinement, and it also provides good matching of the field of the incident wave and the matching surface of the absorber.
- The Fe ground layer promotes internal reflection of photons and blocks transmission, since it plays an active role in the suppression. Suppression of transmission and promotion of photon confinement within the structure are achieved through internal photon reflection, with Fe playing an active role in this process.

A resonator having the resonant dimension in the form of X, intercepted by a resonator having the resonant dimension in the form of Y, closely spans the resonant wavelengths, while previously reported absorbers with uniform resonator dimensions have the resonant wavelength restricted to a particular spectrum or region. This approach also diminishes the spectral gaps of strong absorbers, which are usually present in single-resonance absorbers. The other difference is that the phosphos will respond to a different angle. The proposed configuration is found to show stable absorption for both TE & TM polarisation angles up to relatively high incidence angles. The behaviour is primarily the result of the geometrical symmetry of the circular resonators and the distributed-resonance interaction between the unit cell. Many of these absorbers, to date, based on MXenes, show significant degradation under oblique incidence, either due to the resonators’ anisotropic design or to a lack of impedance match. Also, the present work differs from studies optimising material thickness, in which the mechanisms of resonance distribution are not considered. A parametric study of the dimensions of the circular resonators, the thickness of the dielectric spacer and the parameters of the metallic layers was performed, and the results were analysed. The optimisation was not merely about achieving a single absorption peak; it sought to achieve strong transmission across a wide optical window while maintaining angular stability.

**Fig. 9:**
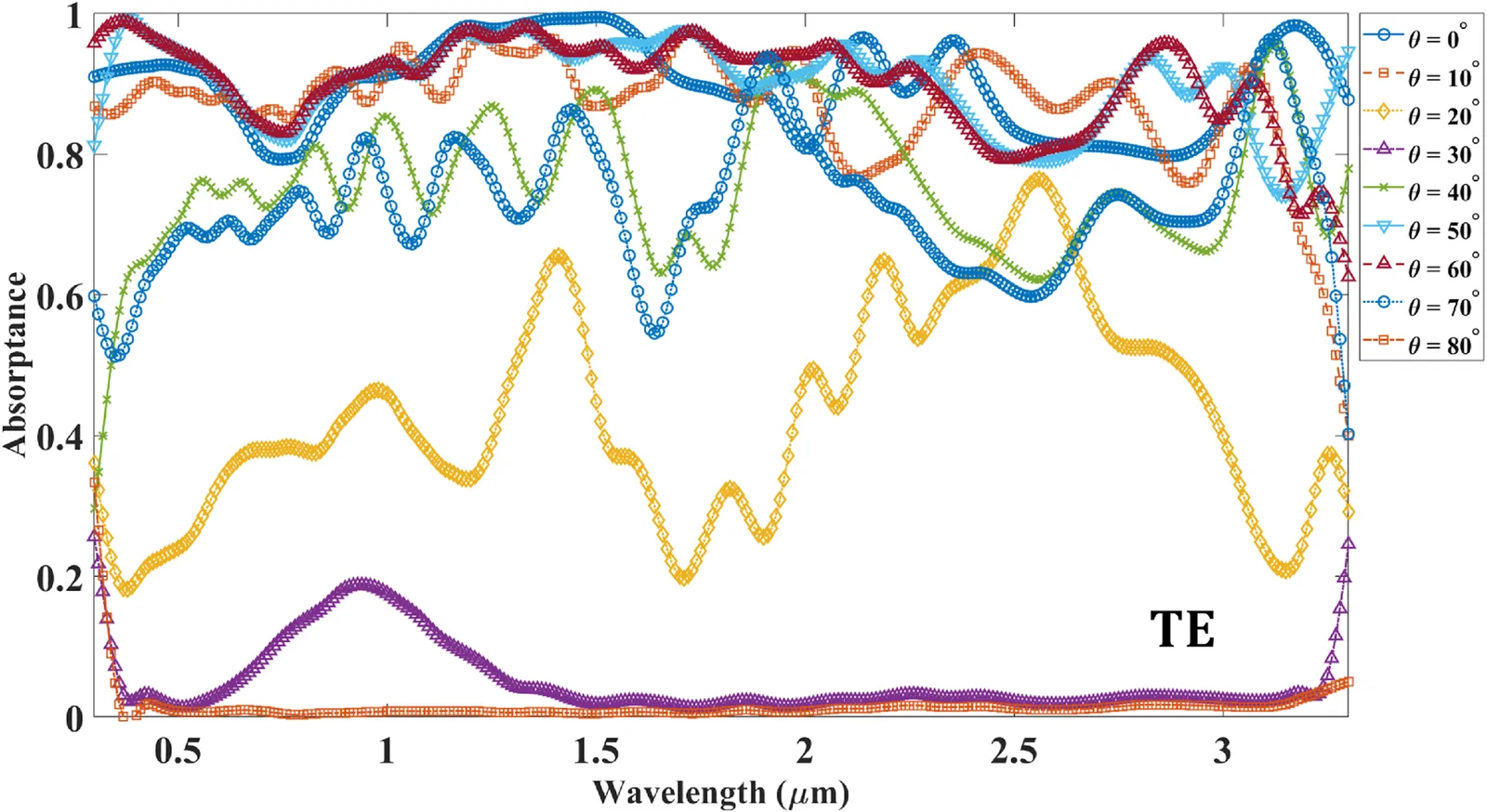
Absorptance spectra under TE polarisation.

Figure 9 shows the absorptance spectra of the structure for TE-polarised light at incident angles ranging from 0° to 80°. At normal incidence (θ = 0°), the absorption is quite high over most of the wavelength range from 0.4 to 3.2 μm, and in many regions it stays above 0.8. This indicates that the structure can absorb light efficiently over a broad range. As the incident angle increases to around 50°, a small reduction in absorption is observed, but the values remain mostly above 0.7. This indicates that the structure does not vary its behaviour to moderate angles. The absorption decreases more visibly and is highly wavelength-dependent when the angle exceeds 50°, particularly at 70° and beyond. The spectra show several oscillations, likely due to resonant and interference effects within the layers. Although performance decreases at very high angles, broadband absorption by the absorber is still observed across a wide range of incident angles with TE polarisation. The decrease in absorption at larger angles can be attributed primarily to an increase in the reflection and a decrease in the strength of the coupling between the incoming light and the structure.

**Fig. 10:**
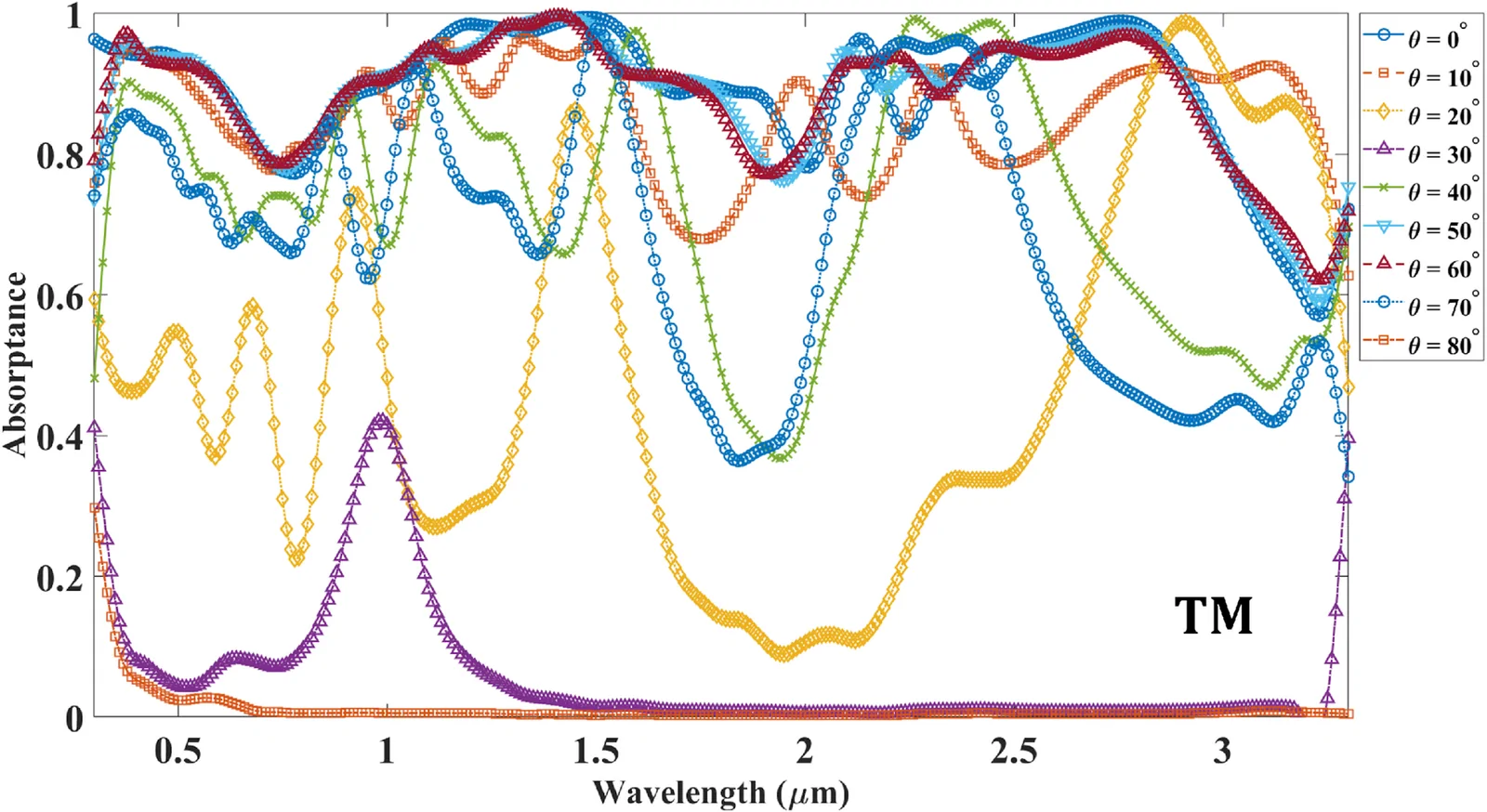
Absorptance spectra under TM polarisation.

Figure 10 presents the spectrograms of the structure’s absorptance as the incident angle is varied from 0 ° to 80 ° under TM-polarised illumination. High values of broadband absorptance above 0.9 are measured at normal incidence (theta = 0) in the wavelength range 0.4–3.2 u, as the wave is highly coupled optically and there is effective impedance matching. The absorptance is relatively constant as the incident angle increases to 500, with only small fluctuations and slight decreases in specific spectral ranges. For angles beyond 50°, particularly for θ ≥ 60°, more pronounced variations and oscillations appear in the absorptance curves, which can be attributed to angular sensitivity arising from changes in the coupling efficiency of TM modes. As can be seen, the absorptance remains greater than 0.8 even at high incidence angles for most wavelengths, and the angle does not strongly affect the absorber’s performance. Multiple resonance modes and plasmonic effects contribute to the broadband and angle-insensitive behaviour, thereby increasing light trapping. The final results suggest that the proposed structure exhibits stable absorption behaviour for the TM polarisation and can be used in photonic, thermal, and energy-harvesting applications, which demand polarisation independence.

**Fig. 11:**
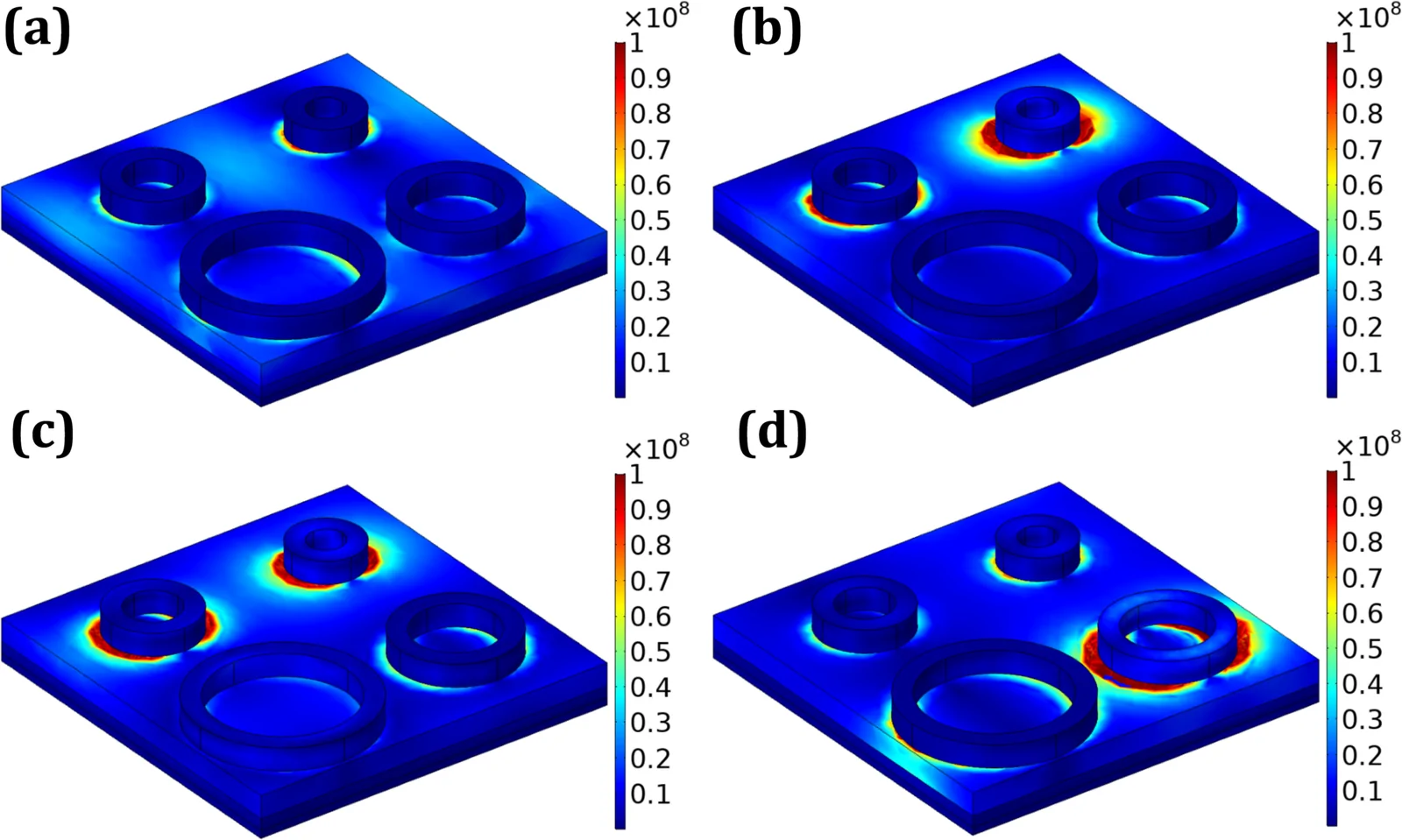
Electric field distributions under TE polarisation (a) 1.5 μm (b) 2.13 μm (c) 2.315 μm (d) 3.17 μm.

**Fig. 12:**
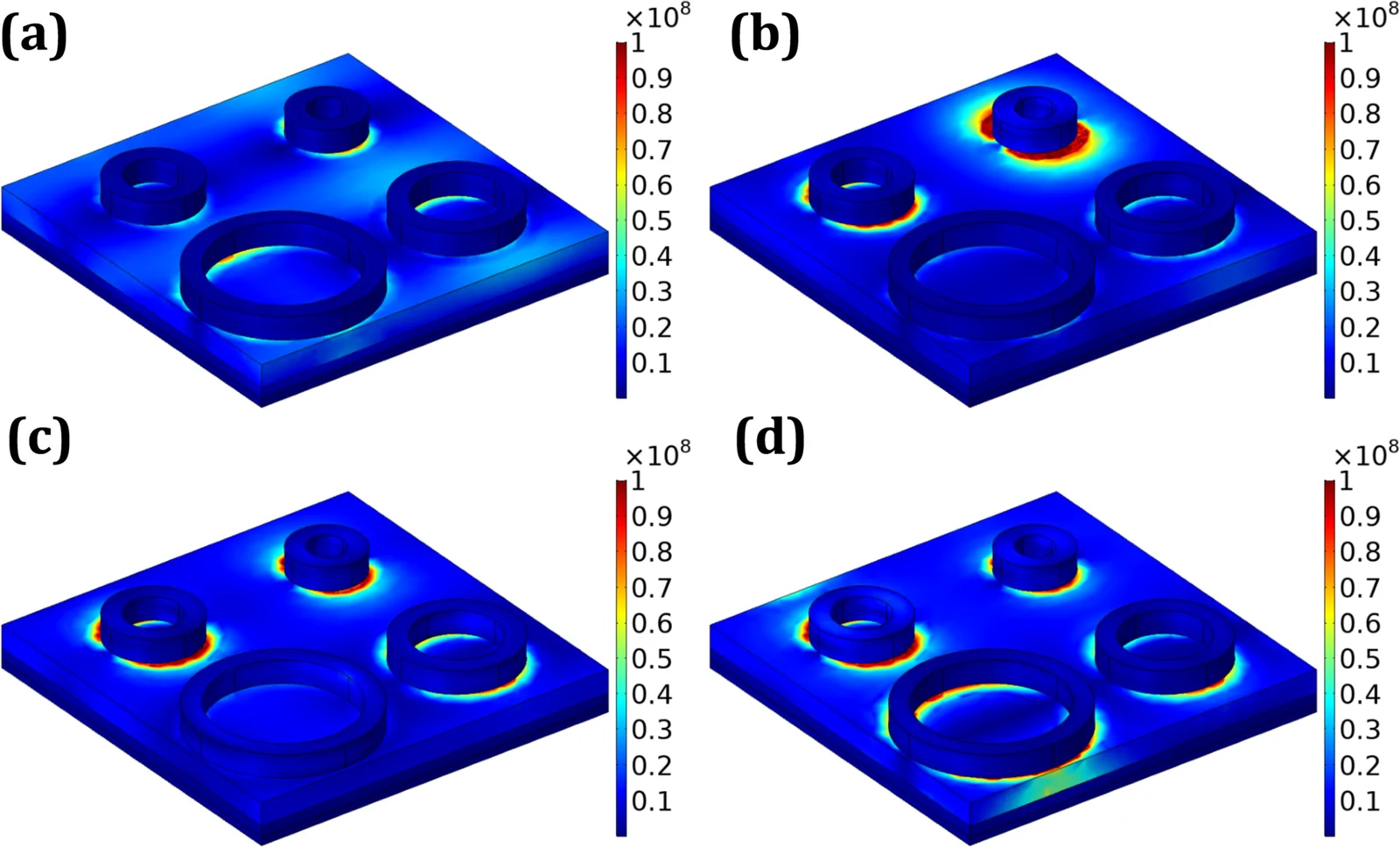
Magnetic field distributions under TE polarisation (a) 1.5 μm, (b) 2.13 μm, (c) 2.315 μm, (d) 3.17 μm.

The selected wavelengths correspond to the major absorption peaks observed in the spectrum: 1.5 μm, 2.13 μm, 2.315 μm, and 3.17 μm, at which the absorptance reaches 0.99, 0.983, 0.985, and 0.98, respectively. These wavelengths were selected because they correspond to different resonance conditions of concentric ring resonators. Figure 11 presents the electric-field distributions. At 1.5 μm, a strong electric-field concentration appears mainly around the largest ring resonator. The field remains localised near the ring edges and dielectric interface, indicating the excitation of localised surface plasmon resonance (LSPR). Since the largest ring has the longest current path, it supports resonance at longer effective optical lengths even within the lower infrared region.

Due to the ring structure, the electric field local intensities are enhanced at 2.13 μm in the upper small ring resonator and, to a lesser extent, in the neighbouring medium-sized ring resonator. This behaviour is a sign of the appearance of coupled resonance between neighbouring metallic rings. This localised enhancement is found near the ring perimeter, further supporting strong charge accumulation and capacitive coupling at the metal–dielectric interface. The simultaneous field localisation appears to be concentrated around two tiny resonators at 2.315 μm. The intensity at the ring gaps and sidewalls becomes even more localised, indicating that multiple resonant modes contribute to it at this wavelength. Such a coupled resonant behaviour is important in ensuring a high absorption coefficient, even in the presence of very slight oscillations in the spectrum.

The field intensity at 3.17 μm is mainly directed towards the larger right-side ring resonator and slightly towards the substrate interface. This means that the longer the path, the more dominant the circulating surface currents are over the resonance at higher wavelengths. This is also reflected in the spatial distribution, with a slightly smoother absorption response in this area.

The respective magnetic-field distributions are displayed in Fig. 12. The magnetic field has been localised in the vicinity of the metallic ring boundaries and between the rings at all wavelengths analysed. The confinement is enhanced below the resonators, showing the existence of anti-parallel current circulation between the metallic resonators and the Fe back reflector, which forms a magnetic dipole resonance. The incident electromagnetic energy is trapped in the resonator layer between the reflector plane, due to the Fe layer’s complete blocking reliability. This process leads to high electromagnetic losses in the MXene and dielectric layers, resulting in near-unity absorption.

The resonator dimensions are also found to relate directly to the resonance behaviour. Larger rings will create resonances at the longer wavelengths due to the longer effective current-path length, while smaller rings produce resonances at the shorter wavelengths. Within a single cell dimension, several different sets of X-rays overlap, indicating the result of a coexistence of several ring sizes. This cooperative response is responsible for the broadband absorption behaviour in the proposed structure.

**Fig. 13:**
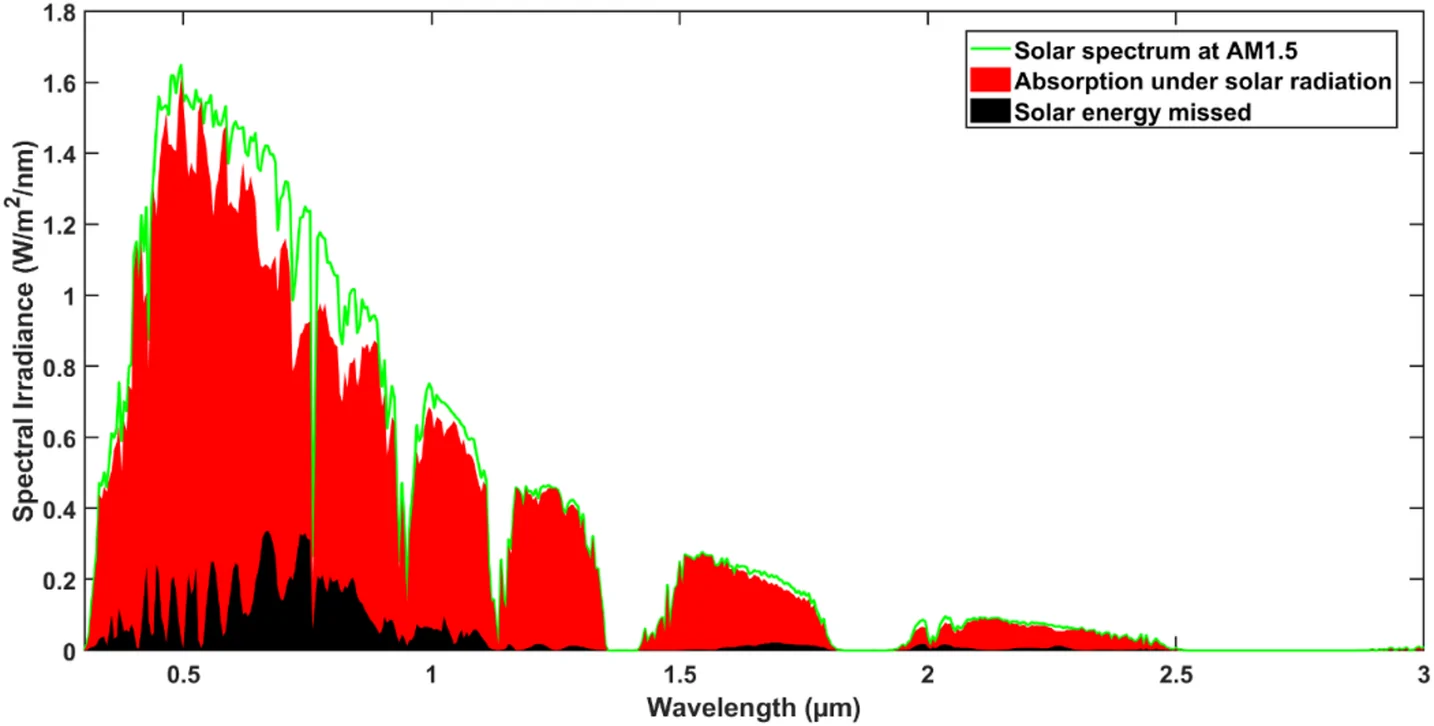
Comparison of spectral AM1.5 solar irradiance.

The absorbance of the Absorber with regard to the standard AM 1.5 solar spectrum is shown in Fig. 13. Scattered sun indicates the sun’s spectral irradiance (green). Absorbed sun is shaded red. Energy not absorbed is shaded black = energy loss. The red and green regions in the middle of the range (0.3–2.5) overlap substantially, suggesting that for the current solar energy, a significant fraction of the existing solar energy will be absorbed by the absorber. High and large absorption capacity confirmed in the visible and near-infrared bands, where the sun’s intensity is higher. Minimal optical losses (as shown by the small black areas) and excellent match with the solar spectrum. This overlap shows that the design is better able to capture solar energy and efficiently convert it into thermal or electrical energy, without energy loss due to reflection or transmission. This essentially means the absorber looks to be a potential candidate for solar harvesting and photothermal applications.

Figure 13 was used to investigate the interaction between the proposed absorber and the incident spectrum over the wavelength range 0.3–3 μm. As can be seen from the plotted response, the designed structure provides sufficient absorption across most of the visible and near-infrared spectrum, with greater intensity under AM1.5. The portion indicated by the shaded region in red corresponds to the absorbed energy from the incident solar energy, and the portion shown in the black shaded region is due to the unabsorbed reflected solar energy from the structure. A response obtained suggests that the circular multi-resonator setup features multiple resonances throughout its spectrum. These modes arise when the size of the metallic ring and the Fe/MXene/SiO2 multilayer configuration differ. The field confinement observed in the electric field distributions also supports this absorption mechanism. Strong localisation around the resonator edges improves electromagnetic energy dissipation inside the structure and reduces reflected power over a broad spectral interval.

In the proposed work, the performance of solar thermal absorbers is generally evaluated using integrated solar absorptance, thermal emissivity, and spectral selectivity. The present work focuses on the optical absorption behaviour of the metasurface absorber rather than on thermal radiation modelling or heat-transfer analysis. The current simulations are restricted to the optical region, whereas thermal emissivity assessment typically requires analysis in the mid- and far-infrared. Figure 13 is intended to illustrate spectral compatibility with the AM1.5 irradiance profile rather than to provide a complete thermodynamic evaluation of a solar thermal device. In the future, work will include mathematical modelling of integrated solar absorptance and infrared emissivity over an extended wavelength range to examine the spectral selectivity and thermal conversion. The proposed absorber contains a continuous Fe ground layer at the bottom of the structure. Due to this metallic backing, the transmission coefficient $\:{S}_{21}$ becomes nearly zero throughout the investigated wavelength range. As a result, the structure does not behave as a freely propagating homogeneous slab in the strict physical sense normally assumed in conventional Nicolson–Ross–Weir type parameter extraction methods.

**Fig. 14:**
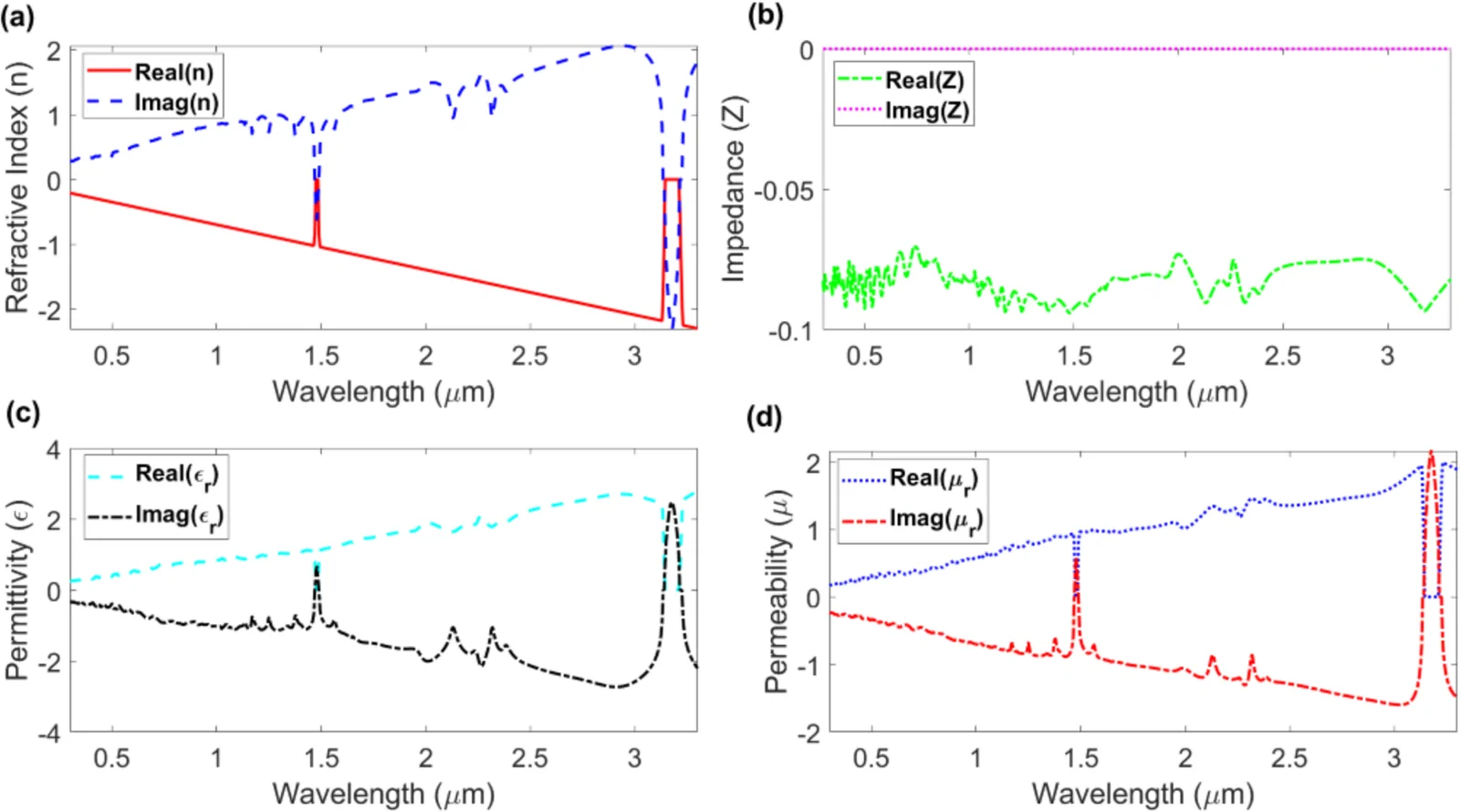
(a) Real and imaginary parts of refractive index with wavelength. (b) Real and imaginary parts of impedance showing resonance-related variation. (c) Extracted permittivity spectra with resonance peaks in both components. (d) Extracted permeability spectra showing a dispersive nature across the operating band.

The extracted parameters in Fig. 14 should therefore be interpreted as effective resonance-related quantities that describe the electromagnetic response of the absorber rather than intrinsic bulk material constants. The retrieval process was performed under the approximation commonly adopted for metamaterial absorbers with metallic back reflectors, where the transmission term is assumed negligible $\:\left({S}_{21}≈0\right)$. Under these conditions, the absorber response is mainly governed by the reflection coefficient S11, and the impedance behaviour becomes the dominant factor controlling absorption performance. To extract the effective relative permeability, permeability$\:\left({\mu\:}_{r}\right)$ nd permittivity $\:{(\epsilon\:}_{r})$ of solar-absorber materials from the S-parameters.

In Fig. 14, the effective electromagnetic parameters extracted from the proposed structure as a function of wavelength, which helps in understanding its resonant and dispersive behaviour. In Fig. 14(a), the real and imaginary parts of the refractive index are plotted versus wavelength. The real part decreases slowly with increasing wavelength, and there is a sharp drop at the resonance wavelength. There are local peaks in the imaginary part, indicating greater material loss and increased interaction between light and the structure at the resonant frequency. These are related to the phase delay, oscillatory effects and absorption. The real and imaginary part of the normalised impedance is given in Fig. 14(b). The real term is close to the matching condition at a large wavelength extent, and the imaginary term is close to zero with relatively small variations. This means they will have good impedance matching at resonance and a low reactive mismatch, leading to high absorption. From a figure like that in 14(c), the permittivity spectra have been extracted with smooth lines for the real part, and sharp peaks in the imaginary part at specific wavelengths. They are the peaks corresponding to the energy loss/destabilisation and energy resonance in the structure. The behaviour of the permeability is plotted in Fig. 14(d), with the real part increasing slowly with wavelength and the imaginary part exhibiting narrow resonance peaks. This is indicative of magnetic resonance effects and dispersive behaviour.

Since the Fe back reflector suppresses transmission almost completely, the contribution of $\:{S}_{21}$is negligible in the evaluated wavelength region. The effective refractive index, permittivity, and permeability were then derived from the retrieved impedance and phase information to study resonance dispersion behaviour inside the structure. To avoid ambiguity, an additional explanation has now been added regarding the thickness definition used during retrieval. The effective thickness corresponds to the total physical thickness of the multilayer absorber stack, including the resonator layer, $\:{\mathrm{S}\mathrm{i}\mathrm{O}}_{2}$ spacer, MXene layer, and Fe reflector. This thickness was used only for phase normalisation during parameter extraction and not to represent a homogeneous bulk medium.

In the present work, the principal branch of the complex phase was selected to maintain continuous wavelength-dependent behaviour of the extracted refractive index. Phase discontinuities caused by resonance transitions were corrected through standard phase unwrapping to avoid nonphysical jumps in the retrieved parameters. This procedure ensured smooth spectral evolution of the electromagnetic quantities over the operating band. Due to the near-zero transmission condition, the extracted constitutive parameters should not be interpreted as exact material constants. In the current paper, their job is primarily to identify regions of resonance and conditions of impedance matching and to determine the dispersive behaviour associated with the absorption enhancement. In particular, firstly, the almost vanishing imaginary component of impedance verifies a minimum reactive mismatch with free space, secondly, the resonance peaks in the imaginary component of the permittivity or permeability imply the presence of electric or magnetic resonators generated by the circular resonators. Thirdly, the dispersive behaviour near the resonant wavelengths attests to the field localisation observed in the electric-field distribution plots.

**Fig. 15:**
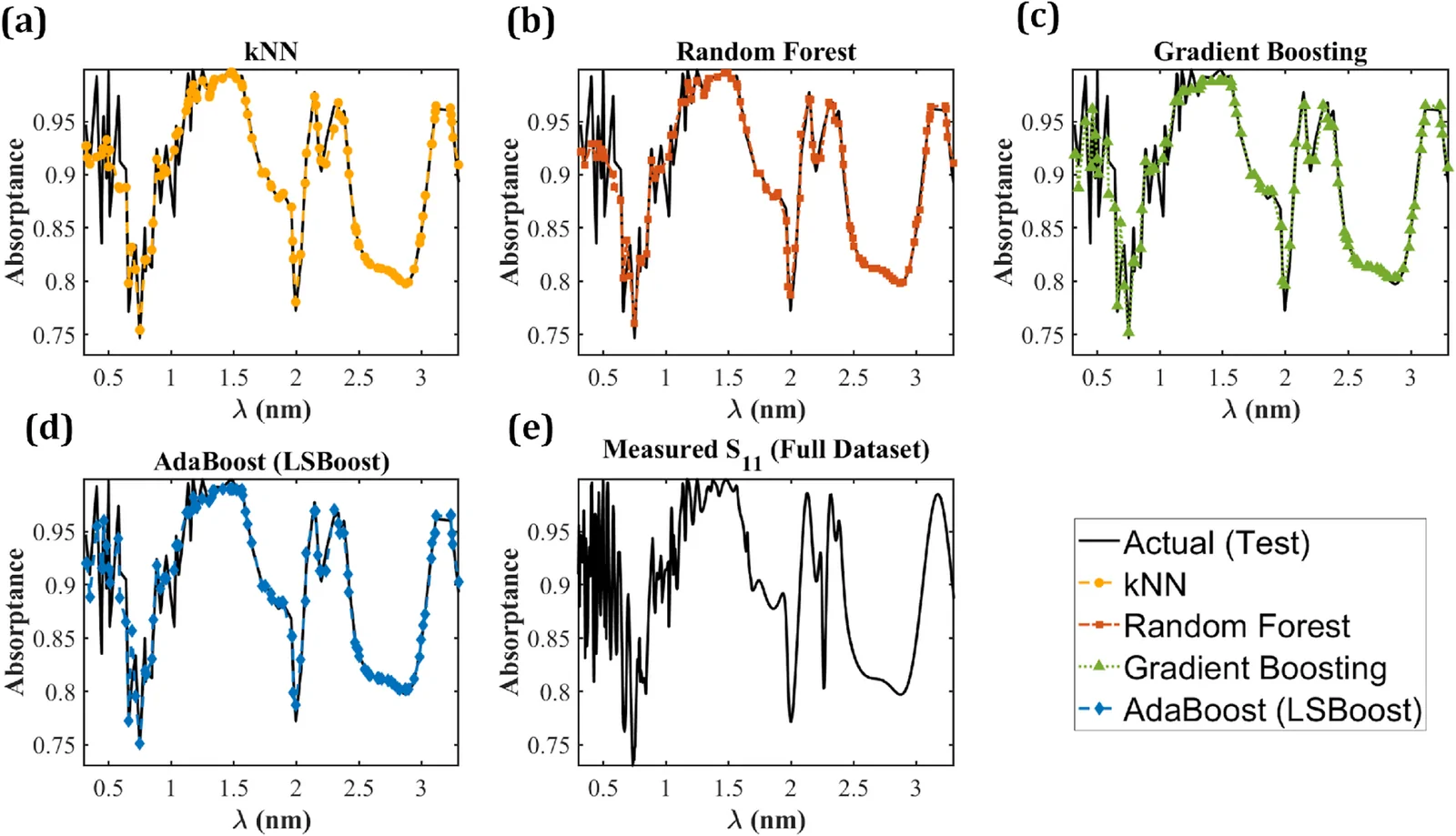
These are comparative absorption spectra of the KNN model, Random Forest, Gradient Boosting, Ada Boost and Actual data.

The ML analysis proved most useful for improving the design and understanding of parameter sensitivity and absorption behaviour as a function of wavelength. The main physical contribution still comes from the absorber configuration itself and the resonance interaction produced by the multilayer circular metasurface arrangement. The proposed absorber differs from earlier MXene-based multilayer absorbers by combining multiple concentric resonators of unequal dimensions with a Metal–SiO_2_–MXene–Fe stack to achieve distributed resonance modes, broad spectral absorption coverage, and stable angular performance under both TE- and TM-polarisation.

The machine-learning study was carried out using supervised regression models trained on the absorption data extracted from finite element method simulations. The input dataset consisted of wavelength values and geometrical parameters of the absorber structure, while the corresponding absorptance values were considered as target outputs. If the input feature vector is represented as per Eq. (6).

6 $$\:\mathrm{X}=\left[\lambda\:,{R}_{1},{R}_{2},{R}_{3},{R}_{4},{h}_{{\mathrm{S}\mathrm{i}\mathrm{O}}_{2}},{h}_{MXene},{h}_{{F}_{e}}\right]$$

Then the regression models estimate the absorptance response. $\:A\left(\lambda\:\right)$ as per Eq. (7).

7 $$\:A\left(\lambda\:\right)=f\left(\mathrm{X}\right)$$

where $\:f(\cdot\:)$represents the nonlinear mapping function learned during training. Before model training, the input parameters were normalised using min–max scaling to reduce numerical imbalance among the variables. The normalisation process is given by Eq. (8).

8 $$\:{X}_{\mathrm{norm\:}}=\frac{X-{X}_{min}}{{X}_{max}-{X}_{min}}$$

where $\:{X}_{min}$ and $\:{X}_{max}$ denote the minimum and maximum values of the corresponding feature. For the K-Nearest Neighbour (KNN) regressor, the predicted absorptance was computed as the weighted average of neighbouring samples in feature space, according to Eq. (9).

9 $$\:\widehat{A}\left(\lambda\:\right)=\frac{1}{K}\sum\:_{i=1}^{K}\:{A}_{i}$$

where * K* represents the number of nearest neighbours and $\:{A}_{i}$ denotes the absorptance value of the neighbouring training samples. In the Random Forest model, the final prediction was obtained by averaging the responses of multiple decision trees, as per Eq. (10).

10 $$\:\widehat{A}\left(\lambda\:\right)=\frac{1}{N}\sum\:_{j=1}^{N}\:{T}_{j}\left(\mathbf{X}\right)$$

where * N* is the total number of decision trees and $\:{T}_{j}\left(\mathrm{X}\right)$ is the output of the $\:{j}^{th}$ tree. This averaging process reduces variance in predictions and improves spectral-fitting stability. For the AdaBoost regression model, the prediction function was constructed by weighted accumulation of weak learners, as in Eq. (11).

11 $$\:F\left(\mathbf{X}\right)=\sum\:_{m=1}^{M}\:{\alpha\:}_{m}{h}_{m}\left(\mathbf{X}\right)$$

where $\:{h}_{m}\left(\mathrm{X}\right)$ represents the weak regression learner and $\:{\alpha\:}_{m}$ denotes its corresponding weight coefficient. The boosting process gradually improves prediction accuracy by assigning larger weights to samples with higher error. Similarly, the Gradient Boosting regressor minimises the prediction loss iteratively through gradient-based correction by following Eq. (12).

12 $$\:{F}_{m}\left(\mathbf{X}\right)={F}_{m-1}\left(\mathbf{X}\right)+{\gamma\:}_{m}{h}_{m}\left(\mathbf{X}\right)$$

where $\:{\gamma\:}_{m}$ is the learning parameter controlling the contribution of the newly added regression tree.

The Neural Network model estimates the absorption response using nonlinear activation between hidden layers. The output of each neuron is represented as per Eq. (13).

13 $$\:y=f\left(\sum\:_{i=1}^{n}\:{w}_{i}{x}_{i}+b\right)$$

where $\:{w}_{i}$ are trainable weights, * b* is the bias term, and $\:f(\cdot\:)$ denotes the activation function. Through repeated weight updates via backpropagation, the network learns the nonlinear relationship between geometric parameters and spectral absorptance. The prediction performance of all models was evaluated using standard regression metrics. The comparative absorption spectra obtained from AdaBoost, Gradient Boosting, KNN, Neural Network, and Random Forest models show that the trained regressors successfully reproduce the broadband spectral behaviour of the proposed absorber. Minor deviations near sharp resonance regions mainly arise from abrupt localised spectral transitions induced by the coupled resonator modes. The ML study confirms that the absorption response of the proposed structure follows a learnable nonlinear relation with wavelength and structural parameters, which supports rapid prediction of absorber behaviour without repeatedly performing computationally expensive full-wave simulations.

Among the regression models used to estimate absorption as a function of wavelength, the model that provides the best fit is shown in Fig. 15, and the data are compared against it. The kNN model tries to follow the general trend of the spectrum in areas with few variations, as shown in Fig. 15(a). Proximity to sharp dips will result in less accurate forecasts, and sharp variations in forecasts are observed in their vicinity. This is because it relies heavily on local data points, which limits its capabilities. The Random Forest result is depicted in Fig. 15(b). The curve is smoother than the one produced by the kNN and is also much closer to the measured values of the principal peaks. Regardless, some minute irregularities become more pronounced, and therefore, a sudden change in the spectrum is not fully appreciated. The Gradient Boosting model agrees well with most wavelengths, as shown in Fig. 15(c). In the high- and mid-absorption regions, there are more peaks; during steep transitions, there are very slight drops that may be localised. Figure 15(d) shows the AdaBoost (LSBoost) outcome. The predetermined curve closely matches the measurements over most of the wavelength range. The resonance depth and the recovery of nonlinear behaviour are reproduced reasonably well, with improved nonlinear behaviour. The measured S11-based absorptance is used as a reference and is displayed in Fig. 15(e). Strong oscillations are observed in shorter wavelengths, and the curve is smooth in longer wavelengths. The boosting-based models adhere more closely to the measured response than the kNN model, particularly in areas where the spectrum varies quickly.

**Fig. 16:**
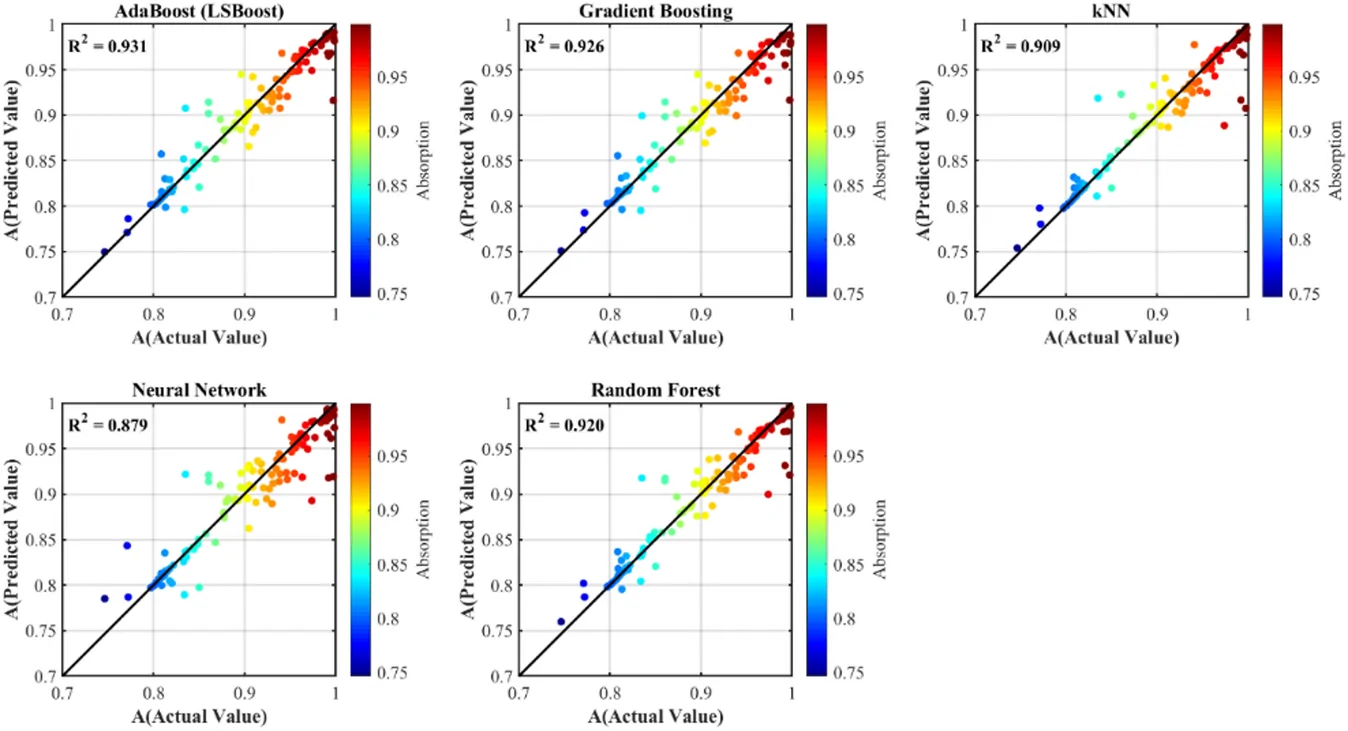
Comparative absorption spectrum of Different ML models: (a) Ada Boost, (b) Gradient Boosting, (c) KNN, (d) Neural Network, (e) Random Forest.

The results are shown in Fig. 16, which compares predicted and measured values. The diagonal line indicates perfect agreement between the predicted and measured results. Figure 16(a) shows that the AdaBoost (LSBoost) model places most data points close to this line, i.e., the predictions are very close to the measured values. The good prediction across the entire range of absorptance is indicated by an R2 value of 0.931, and it is especially good at higher absorptance values exceeding 0.9. The Gradient Boosting results are presented in Fig. 16(b). The same trend is observed, with R2 = 0.926. At medium levels of absorptance, some spread of points may be observed, but the overall match is good. Figure 16(c) also shows that the kNN model yields a lower R^2 value of 0.909. The scatter is also more pronounced, especially at lower absorptance values, indicating that the model is sensitive to the distribution of the data points. Figure 16(d) the Neural Network results. Here, deviations greater than the diagonal line are observed, resulting in an R^2^ of 0.879. This implies that the current dataset does not fit it as well. Figure 16(e) the Random Forest model that provides a balanced performance with a value of R^2^ of 0.920. Averaging helps reduce prediction errors, and certain sharp transitions are not perfectly sharp. Table 1 compares different multilayer absorber designs. The results show that the proposed structure provides strong broadband absorption and maintains stable performance over a wide range of incident angles and wavelength bands.

**Table 1:** Different multilayered absorber structures.

Refs.	Abs. η (%)	Band	Size (nm^3^)	Angle Stability	Layers
Pro.	< 90	0.87–1.79 2.07–2.425 3.045–3.295	1200 × 1200 × 425	60°	Fe/Sio_2_/Mxene/Fe
^24^	98	0.3–3.0	200 × 2000 × 1050	8°	Tungsten/MgF_2_/Gold
^25^	93.17	0.2–1.9	500 × 500	45°	Au / SiO_2_ / W / Tio_2_
^26^	95.35	0.2–2.5	1200 × 1200 × 740	5°	TiO_2_ / Al_2_O_3_ / ZrO_2_ / Graphite
^27^	≤ 90	0.3–1.5	1000 × 1000 × 310	7°	Au / SiO_2_
^28^	99.99	0.3–0.6	500 × 500	6°	Tungsten / SiO_2_
^29^	98.8	0.4–0.5	500 × 450 × 60	6°	SiO_2_ / Tungsten / Si_3_N_4_
^30^	98.1	0.7–1.4	400 × 400 × 820	6°	Fe / InSb / Al
^31^	≤ 80	0.2–2.0	90 × 90 × 290	7°	MXene / AlN
^32^	99.99	9.9–10.0	150 × 1700 × 325	6°	Au / TiO_2_
^33^	96	0.2–3.0	240 × 170 × 230	6°	TiN / TiO_2_
^34^	78	0.3–20	1200 × 1200 × 420	8°	W / MgF_2_ / Ti
^35^	41	1.0–3.9	383 × 383 × 110	9°	W / AlN
^36^	99.99	0.4–0.5	520 × 520 × 259	9°	Au / GaAs
^37^	91.8	0.3–2.2	509 × 200 × 88	7°	SiO_2_ / TiO_2_ / W
^38^	99.99	0.6–0.8	420 × 420 × 155	9°	Al / GaAs
^39^	99.8	0.3–1.1	190 × 190 × 300	6°	Ti / SiO_2_ / Al
^40^	> 98	0.2–2.9	2000 × 2000 × 3830	35°	Au / MgF / Tungsten
^41^	99.96	0.4–0.5	566 × 566 × 260	9°	Al / GaAs

## Conclusion

The article presents a multilayered solar absorber structure for solar harvesting applications. The proposed structure shows more than 90% absorption over 0.87–1.79 μm, 2.07–2.425 μm, and 3.045–3.295 μm. The overall size of the structure is 1200 × 1200 × 425 nm^3^. Various variations, such as changes in resonator size, filled and engraved behaviour, and height changes in the resonator, substrate, Mxene, and ground layers, were implemented to achieve peak absorption. The proposed structure shows more than 90% absorption over 0.87–1.79 μm, 2.07–2.425 μm, and 3.045–3.295 μm. The TE and TM mode behaviour with variations in incident angle was investigated with respect to various performance metrics. The proposed design represents 60° degrees of angle stability. The performance of the proposed design is compared with different machine learning models. The broadband absorption spectrum is best suited for thermal-harvesting applications.

## Data Availability

Data will be available form the corresponding author based upon reasonable request.
